# Knockdown Experiment Reveals an Essential GTPase CgtA’s Involvement in Growth, Viability, Motility, Morphology, and Persister Phenotypes in Vibrio cholerae

**DOI:** 10.1128/spectrum.03181-22

**Published:** 2023-03-14

**Authors:** Sagarika Das, Ananya Chatterjee, Partha Pratim Datta

**Affiliations:** a Department of Biological Sciences, Indian Institute of Science Education and Research Kolkata, Nadia, West Bengal, India; Post Graduate Institute of Medical Education and Research

**Keywords:** *Vibrio cholerae*, CgtA, GTPase, gene knockdown, proteomics, microbiology

## Abstract

CgtA is an essential bacterial GTPase consisting of a highly conserved N-terminal Spo0B-associated GTP-binding protein (Obg) domain, a central GTPase domain, and a variable C-terminal domain (CTD). This study reports global changes in the proteome and transcriptome of wild-type (Wt) versus full-length CgtA-depleted Vibrio cholerae in minimal media. Comparative transcriptome sequencing (RNA-Seq), followed by comparative proteomic analyses, revealed that the knockdown of *cgtA* significantly altered expressions of 311 proteins involved in diverse cellular activities, many of which are associated with the survival of V. cholerae. Various intracellular functional roles of CgtA in growth, viability, motility, morphology, and persister phenotypes in V. cholerae are revealed based on subsequent confirmatory experiments. Furthermore, a more sustained mRNA expression pattern of *cgtA* in a minimal medium than in a rich medium was also observed for Wt V. cholerae, where the highest level of mRNA expression of *cgtA* was observed during the logarithmic growth phase. Thereby, we propose that minimal medium-associated reduced growth rate coupled with *cgtA* depletion aggravates the intracellular stress in V. cholerae, interrupting vital cellular processes. The functional role of the CTD in V. cholerae is not fully understood. Hence, to specifically investigate the intracellular role of the 57-amino-acid-long CTD of CgtA_VC_, the CTD-only portion of CgtA was deleted. Subsequent proteomics studies revealed an altered expression of 240 proteins in the CgtA(ΔCTD) mutant, having major overlap with the full-length *cgtA*-deleted condition. Overall, our study reveals an alternative facet of the survival mechanism of V. cholerae during nutritional downshift as per the concomitant consequences of *cgtA* depletion.

**IMPORTANCE** It is very important that we must find new drug target proteins from multidrug-resistant human-pathogenic organisms like V. cholerae. CgtA is among such potential candidates, and here, we are reporting about some newly identified cellular roles of it that are important for the survival of V. cholerae. Briefly, we knocked down the full-length *cgtA* gene, as well as did a partial deletion of this gene from the V. cholerae genome followed by RNA-Seq and proteomics studies. Results from our study revealed up- and downregulation of several known and unknown genes and proteins as the effect of the *cgtA* knockdown experiment. Also, we have presented some interesting observations that are linked with *cgtA* for growth, viability, motility, morphology, and persister phenotypes in V. cholerae. Our study enhances the importance of CgtA and paves the way for further exploration based on our provided data.

## INTRODUCTION

Bacteria adopt a multitude of adaptive response mechanisms to encounter various environmental stresses. Vibrio cholerae, a motile Gram-negative pathogenic bacterium, modifies its physiology to withstand diverse stresses like nutritional stress, osmotic stress, thermal stress, and acid stress while transitioning its habitat between aquatic environments and the gut of the human host (1). Adaptation to nutritional starvation in V. cholerae is associated with morphogenesis into viable but nonculturable (VBNC) or persister cells, biofilm formation, quorum sensing, and elevated levels of the alarmone (p)ppGpp, leading to optimization of global gene expression. (p)ppGpp binds to its molecular targets, DksA and RNA polymerase, to affect the virulence and transcriptional machinery of V. cholerae during nutritional stress. In V. cholerae, the intracellular (p)ppGpp levels are modulated by the key cellular proteins RelA, RelV, SpoT, DksA, and CgtA. RelA and SpoT are the two major bifunctional (p)ppGpp synthetase/hydrolases. RelA is primarily associated with translating ribosomes and is activated during amino acid starvation, leading to stringent response elicitation (2). RelA possesses a predominant (p)ppGpp synthetase activity, whereas SpoT possesses a weak (p)ppGpp synthetase and a strong (p)ppGpp hydrolase activity. SpoT is associated with pre-50S ribosomes (2) and is involved in carbon, fatty acid, and phosphate starvation in bacteria (3). RelV is a single-domain protein with (p)ppGpp synthetase activity during glucose and fatty acid starvation (4).

For bacteria, nutrient-rich conditions (for example, lysogeny broth [LB] media) are nonstressful conditions, whereas limiting the bacteria for nutrients elicits stress. A critical role of SpoT in V. cholerae is attributed to repressing the stringent response under nutrient-rich conditions when the growth rates are higher than under nutrient-limiting conditions. There is a steady basal-level increment of ppGpp levels in the cells in the nutrient-rich media since ppGpp is not efficiently degraded. SpoT degrades the basal levels of (p)ppGpp that might elicit the stringent response in cells (5). CgtA is a conserved and essential GTPase in bacteria that acts as a cellular GTP/GDP pool sensor. CgtA belongs to the translation factors (TRAFAC) class of P-loop GTPases. CgtA is implicated in cell growth, development, initiation of sporulation, protein translation, and chromosome segregation (6). The diversity in the functionality of CgtA GTPases across bacterial niches is evident from the variations in the GTP hydrolysis rate of the CgtA from various bacteria (7). Caulobacter crescentus CgtA is associated with free 50S ribosomes instead of 70S or translating ribosomes (8). Also, the C-terminus of CgtA is vital for the 50S association (8). The *in vitro* GTPase activity of wild-type (Wt) CgtA is more highly elevated when coupled to purified 50S ribosomes than its intrinsic state (9). In Escherichia coli, mutations in CgtA are associated with rRNA processing and polysome defects (10). It is reported that *cgtA* is coexpressed from an operon encoding two ribosomal proteins (L21 and L27) (11). The operon expression is variable with the growth phase and regulated by the transcriptional regulators DksA and ppGpp (11). CgtA is considered a specialized stress response-associated translation factor as augmented interaction of CgtA with 50S ribosomes is observed upon binding to ppGpp (12).

The depletion of CgtA in V. cholerae under nutrient-rich conditions is accompanied by the global changes in mRNA expression repertoire, similar to classical low-nutrient stress response (stringent response) associated with elevated (p)ppGpp levels (5).

In E. coli, it is reported that CgtA predominantly associates with the 50S ribosomes and interacts with SpoT (13). The functional activity of SpoT is regulated by CgtA (5). CgtA functions by repressing the stringent response to maintain low alarmone (ppGpp) levels, especially when V. cholerae grows in a nutrient-rich environment by interacting with SpoT. Thus, CgtA suppresses the stringent response under nutrient-rich growth conditions (5). Thus, in V. cholerae, CgtA can be considered a bonafide nutritional stressor.

Under the unstressed condition (nutrient-rich media), bacterial cells are likely to focus more on growth and replication (14). In contrast, natural growth is compromised under nutritionally stressed conditions (minimal media). The ongoing molecular and cellular processes are highly engaged when bacteria undergo rapid growth under nutrient-rich conditions. These processes include protein translation, rapid DNA replication, cell division, and cellular reorganization (15). It is reported that bacterial cells demonstrate uncoupling between cell division and DNA replication and segregation every 20 to 40 min under optimal conditions. As chromosomal DNA is highly condensed and ~1,000 times the cellular length, the segregation of DNA requires a longer time (60 to 90 min) (15). A temperature-sensitive mutation in the CgtA of E. coli revealed a defect in nucleoid partitioning accompanied by cell elongation (6). Also in E. coli, the ectopic expression of DnaA levels could rescue DNA replication defects arising in a temperature-sensitive mutation (7). Thus, CgtA controls chromosome partitioning and DNA replication. Hence, the consequential role of CgtA in cell division in V. cholerae under nutrient-rich or -limiting conditions could be reiterated by the morphological studies of the cells depleted of full-length CgtA or C-terminal domain (CTD)-truncated CgtA, designated, in this study as CgtA(ΔCTD).

The current information available on the CgtA of V. cholerae is based on nutrient-rich growth conditions. In this current study, we decipher the role of CgtA in V. cholerae by exposing the cells to a nutrient-limiting environment (minimal media) and compare the effects by growing in nutrient-rich (LB) media. Thus, a fair scope exists to explore the functional roles of CgtA under nutrient-limiting growth conditions. It is important to investigate the role of full-length CgtA when the carbon sources are limited in media during growth by predepleting the intracellular levels of CgtA gradually from a *cgtA* knockdown strain. Following a similar approach, the *in vivo* role of a 56-amino-acid-long CTD-truncated CgtA strain is deciphered in this study. The strains were constructed by a two-step allele replacement strategy by homologous recombination. The effects on viability, growth patterns, doubling times, motility, morphology, and outcomes of continual nutritional stress on the emergence of persister phenotype are presented in this study upon full-length CgtA depletion and CgtA(ΔCTD) conditions. In order to gain deeper insights into the cellular networks associated with CgtA GTPase, the enrichment of various interconnected pathways was achieved by using high-throughput techniques, including genome-wide transcriptome analysis by transcriptome sequencing (RNA-Seq) and label-free comparative proteomics of the *cgtA* knockdown and CgtA(ΔCTD) compared to the Wt V. cholerae.

## RESULTS

### CgtA aids in the adaptation of physiologic responses during nutrient-limiting conditions.

**FIG 1:**
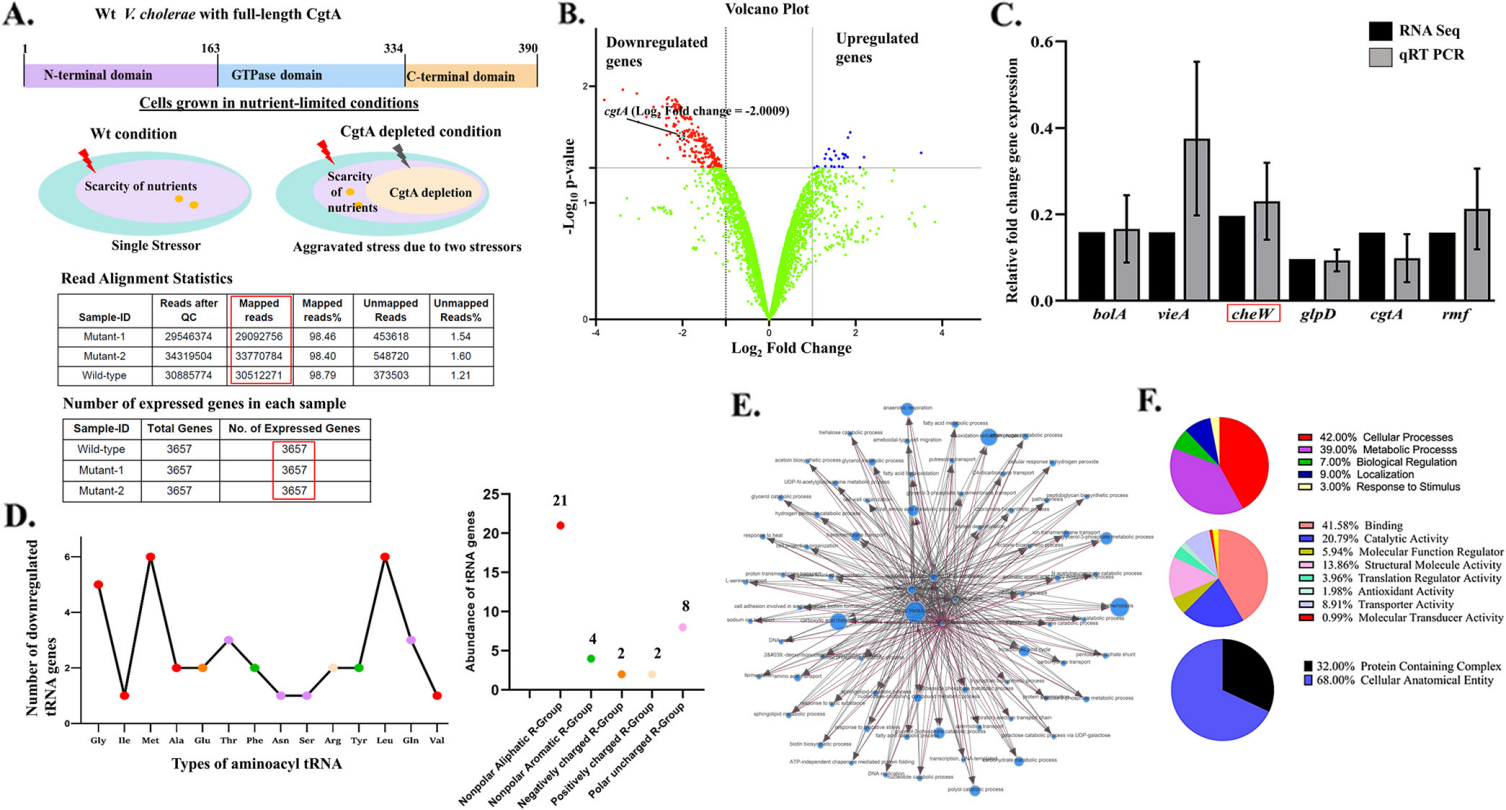
(A) Hypothesis of the study, read alignment statistics of RNA-Seq, and the number of expressed genes in each sample. (B) Volcano plots showing the up- and downregulated genes in full-length CgtA-depleted cells versus Wt cells in M9 minimal media. (C) Validation of differential gene expression of six genes obtained by RNA-Seq by qRT-PCR. The graph represents a comparison of the relative gene expression in both the RNA-Seq analysis and qRT-PCR experiments. (D) tRNA genes downregulated in Δ*cgtA::kan^r^* V. cholerae. (E) Network analysis to predict the pathways and interactions of DEGs that were predicted under the CgtA-depleted condition. (F) Gene ontology (GO) analysis.

In order to explore the diversity of physiological responses that actively participate in V. cholerae during nutrient-limiting conditions (Fig. 1A), we first performed RNA-Seq in Wt V. cholerae to identify genes whose expression levels were differentially regulated compared with the V. cholerae with depleted levels of the essential GTPase, CgtA. Essential proteins modulate the adaptive physiological responses (16) when metabolic activities are compromised, while the expression level of the most functional genes is diminished. Based on RNA-Seq data, we identified the differentially expressed genes under full-length CgtA-depleted conditions compared with Wt cells in M9 minimal media harvested from mid exponential phase. A total of 228 genes were downregulated; the fold change of expression ranged from ~2- to 10-fold, whereas 26 genes were upregulated, and the fold change of expression ranged from ~2.4- to 4.2-fold (Fig. 1B). Therefore, the proteins encoded by these genes are expected to be regulated directly or indirectly by CgtA to aid in adapting physiologic responses arising from nutrient limitation. The genes encoding chemotaxis proteins, membrane transporters, signal transducers, stress response proteins, transcriptional regulators, and metabolic proteins are the most downregulated. A few downregulated genes obtained by RNA-Seq were validated by reverse transcription-quantitative PCR (qRT-PCR). A graph depicting a comparison of the relative gene expression in both the RNA-Seq analysis and qRT-PCR experiments was plotted (Fig. 1C). All of the selected genes for validation by qRT-PCR, namely, *rmf*, *vieA*, *cheW*, *bolA*, *glpD*, and *cgtA*, were significantly downregulated (both in terms of fold change of expression and *P* value) under the *cgtA* depletion (knockdown) condition. The *rmf* gene encodes ribosome modulation factor (RMF). It is less expressed in slow-growing cells. RMF is involved in the formation of 100S ribosomes in the stationary phase. *vieA* encodes a response regulator protein to regulate biofilm formation and flagellar motility. *bolA* is a DNA binding transcriptional regulator and general stress response protein and is involved in cell division. *cheW* is involved in chemotactic motility and works as a scaffolding protein in chemotactic arrays. Of all the significantly downregulated genes under the *cgtA* knockdown condition, *glpD* was the most downregulated, with a fold change of 0.09620. *glpD* encodes glycerol 3-phosphate dehydrogenase, which catalyzes the oxidation of glycerol 3-phosphate to dihydroxyacetone phosphate.

Interestingly, tRNA downregulation was also observed from RNA-Seq data during the *cgtA* knockdown condition (Fig. 1D). During stressful conditions, cells alter their tRNA abundance to selectively regulate the translation of proteins (17). Hence, the observed phenomenon could be due to the rearrangement of the tRNA pool due to the general stress response arising from *cgtA* depletion in V. cholerae cells to influence protein synthesis. The altered transcriptome results from aggravated stress due to *cgtA* depletion and growth in a minimal media (Fig. 1A).

Through network analysis, the pathways and interactions of differentially expressed genes (DEGs) were predicted under the *cgtA*-depleted condition (Fig. 1E). The gene ontology (GO) analysis revealed various molecular functions and biological processes that affected CgtA depletion (Fig. 1F).

On the contrary, ΔCTD *cgtA::kan^r^* showed a negligible DEG pattern (see Fig. S1 in the supplemental material).

**FIG 2:**
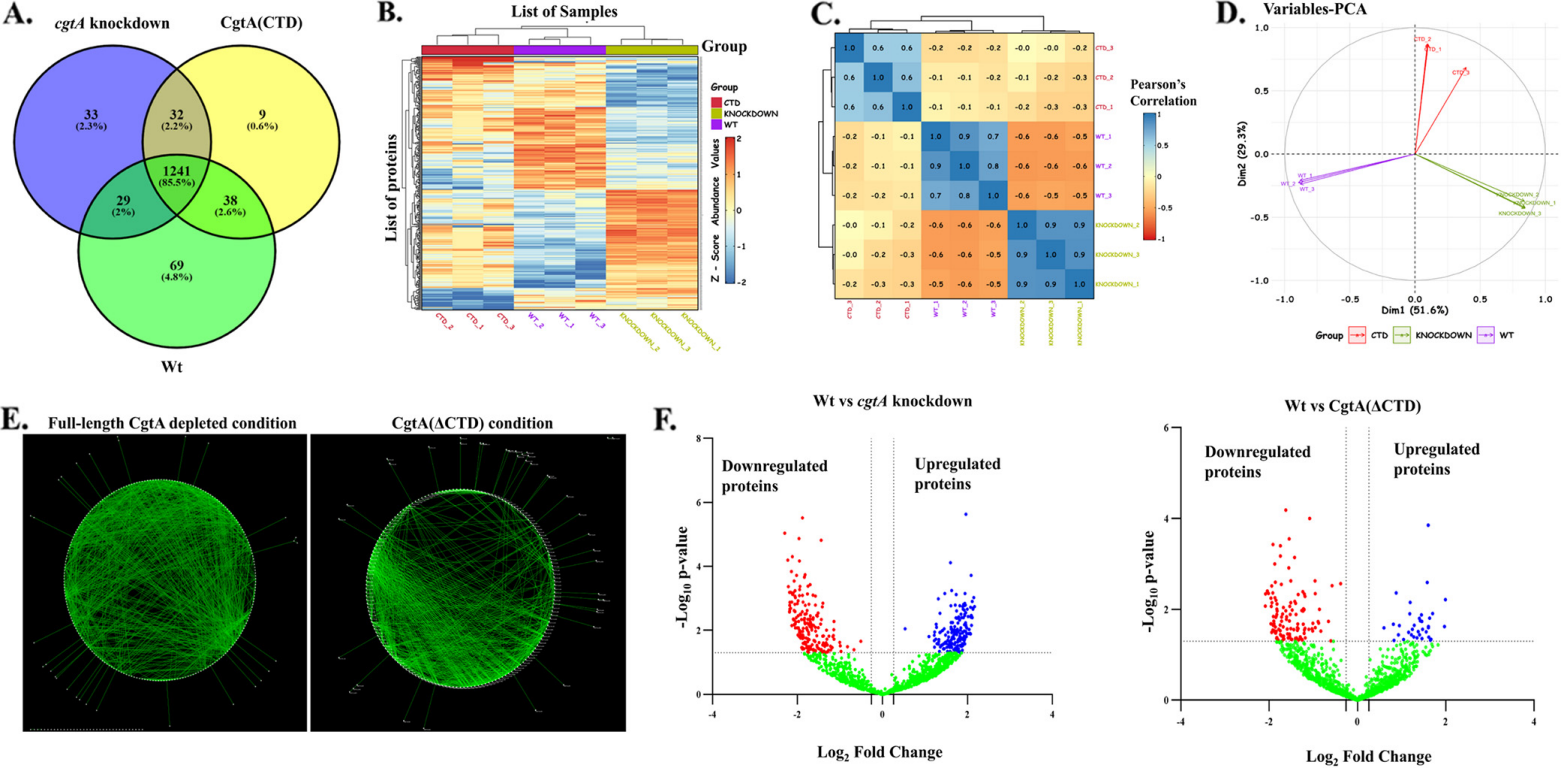
(A) Venn diagram showing the number of proteins identified under each of the conditions and also the proteins common between them. (B) Heatmap showing the ANOVA-significant proteins under all three conditions. (C) Correlation matrix between all the groups. (D) Principal-component analysis (PCA) showing clusters of all the samples based on their similarity. (E) Protein-protein interaction network of ANOVA-significant proteins under both full-length CgtA depletion and CgtA(ΔCTD) conditions. (F) Volcano plots showing significant up- and downregulated proteins.

The results from global proteomic analyses also revealed critical information along similar lines. The total number of proteins identified for each of the three strains are as follows: 1,377 for Wt, 1,335 for *cgtA* knockdown, and 1,320 for CgtA(ΔCTD). The total number of proteins common to each strain is represented in the Venn diagram (Fig. 2A). The proteins identified by label-free proteomics were statistically validated by the analysis of variance (ANOVA) statistical test. The proteins upregulated and downregulated were represented through a heatmap (Fig. 2B). The magnitude of the protein expression levels of the ANOVA-significant proteins are depicted as colored grids. The Z score of protein abundance values ranged from −2 to +2. A correlation plot (Fig. 2C) identifies the degree of similarity between the samples based on Pearson’s correlation coefficient. Principal-component analysis (PCA) for the proteomics data is shown (Fig. 2D).

Of the 311 altered proteins under the CgtA-depleted condition (after applying both *P* value and fold change cutoff filters), 280 proteins were used for network analysis (Fig. 2E). The remaining 31 proteins were functionally uncharacterized. Out of the 240 altered proteins under the C-terminal domain CgtA-deleted condition, 209 proteins were used for network analysis (Fig. 2E). The rest of the 31 proteins were functionally uncharacterized. Compared to the high-density protein-protein interaction (PPI) network prevalent under the *cgtA*-depleted condition, a relatively lower-density protein-protein interaction network is observed under the CgtA(ΔCTD) condition (Fig. 2E). A pairwise comparative study of the altered proteomic profiles between a deletion mutant and Wt strain is represented. Instead of an ANOVA statistical test, a *t* test was performed to calculate the *P* values. Volcano plots were used to visualize the alterations in the proteome. A volcano plot is a scatterplot that plots the negative log_10_ *P* values (statistical significance) versus the log_2_ fold change. The cut-off value of the *P* value is 1.3 on the volcano plot [since negative log_10_(0.05) = 1.3]. The negative log_10_ *P* value and log_2_ fold change cutoff values are represented as dotted lines on the *y* axis and *x* axis, respectively, in the volcano plot. All the proteins with a negative log_10_ *P* value above 1.3 are significant (colored red and blue). The nonsignificant proteins are green in color. In order to segregate the statistically significant proteins based on the fold change, proteins with fold change between +0.26 and −0.26 are not considered. The most significant proteins appear skewed at the top of the plot. The upregulated proteins appear in the top right-hand side panel of the volcano plot (blue colored), and the downregulated proteins appear in the top left-hand side of the volcano plot (red colored) (Fig. 2F). In group 1, the proteome of the *cgtA* knockdown (KD) strain is compared with the Wt. In group 2, the CgtA(ΔCTD) proteome is compared with Wt. In group 1, upregulated proteins (colored red) are more abundant than in group 2. Also, the number of downregulated proteins (colored blue) is comparatively higher than in group 2.

It is crucial to understand which critical biological processes are highly affected when the V. cholerae cells are depleted of full-length CgtA. A bidirectional bar chart is plotted to highlight the cellular processes in which the number of proteins is highly altered (Fig. S2). The number of proteins significantly downregulated (red-colored bar) and significantly upregulated (blue-colored bar) can be visualized along with the biological processes. Fifty-four biological processes are disrupted in the Δ*cgtA* knockdown strain of V. cholerae. The top 10 altered biological processes involving the maximum number of altered proteins are highlighted in decreasing order. A comparative presentation of up- and downregulated proteins of some critical metabolic pathways for full-length CgtA knockdown and CgtA(ΔCTD) conditions is shown in Fig. S3.

### CgtA plays a critical role in supporting the growth and viability of V. cholerae in minimal media.

**FIG 3:**
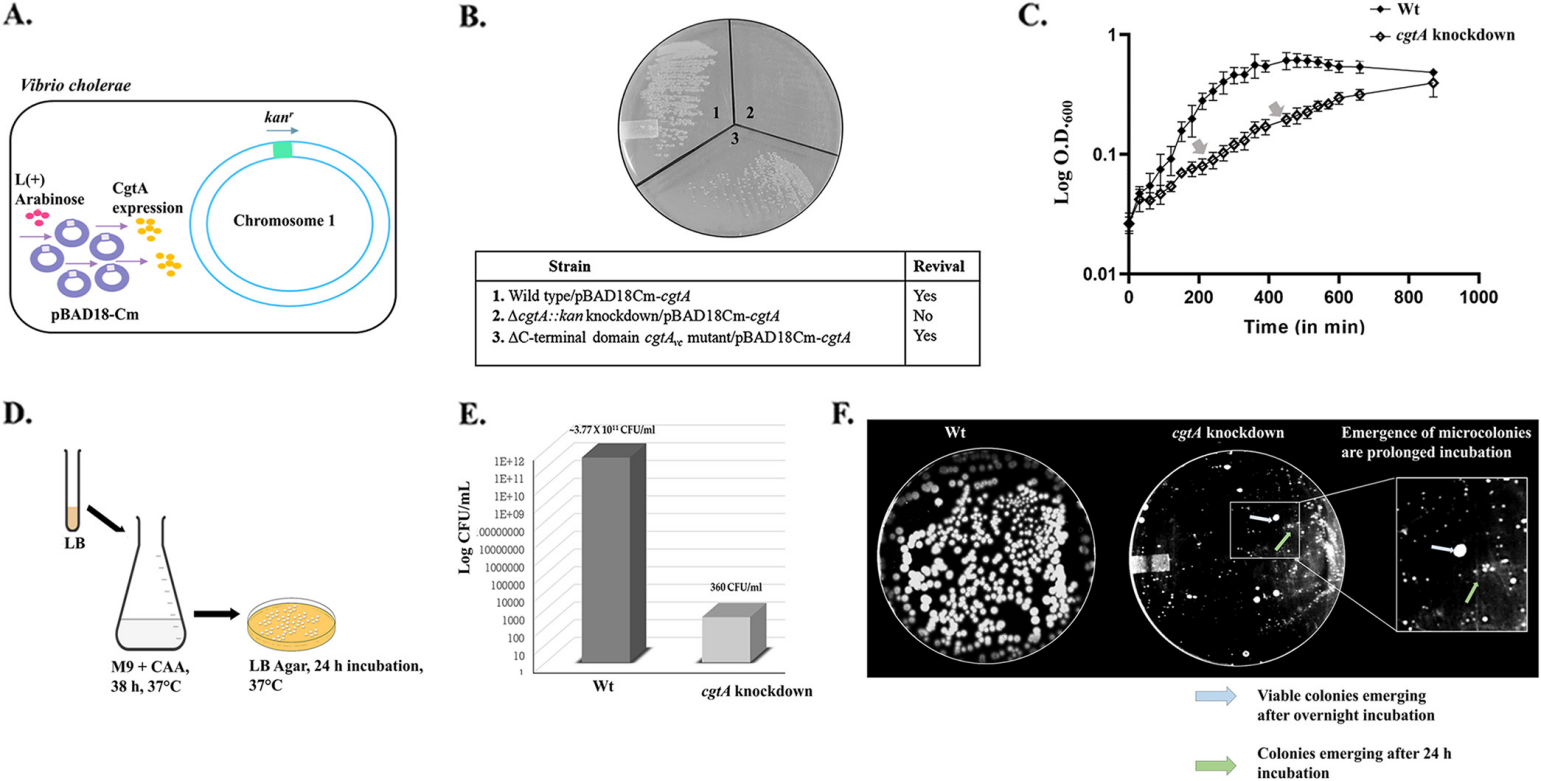
(A) Illustration of full-length *cgtA* knockdown in V. cholerae while expression of full-length CgtA from pBAD18Cm vector was under the influence of an arabinose inducer. (B) V. cholerae strain with *cgtA* gene knockdown could not be revived from −80°C glycerol stock when arabinose was not supplemented on an LB agar plate, whereas the Wt and CgtA(ΔCTD) could be revived without arabinose supplementation. (C) Growth pattern of Wt versus full-length *cgtA* knockdown in M9 minimal media. (D) Estimation of log CFU/mL. (E) Evidence of the resuscitation of persister cells upon withdrawal of nutritional stress.

Wt *cgtA* was deleted by replacing the wild-type *cgtA* open reading frame (ORF) with a kanamycin marker while expressing Wt *cgtA* from the pBAD18Cm-*cgtA* in the presence of arabinose (Fig. 3A). Similarly, the CgtA(ΔCTD) strain was constructed by replacing the Wt *cgtA* with the *cgtA*(ΔCTD) allele fused to the kanamycin marker.

When the CgtA(ΔCTD) freezer stock was streaked on LB agar plates, the strain could grow without arabinose supplementation in the media (Fig. 3B). On the contrary, the *cgtA* knockdown strain could not be revived from the freezer stocks when streaked on LB agar plate without arabinose supplementation (Fig. 3B). Thus, depletion of CgtA has a deleterious effect on the survival of V. cholerae. In contrast, the deletion of the C-terminal domain does not affect the survival of V. cholerae. Hence, it is implied that the essentiality of the V. cholerae CgtA GTPase lies in the N-terminal domain and the GTPase domain, but not in the C-terminal domain.

The growth of *ΔcgtA::kan^r^* was severely affected compared to Wt. The *cgtA* knockdown strain grew for 12 h before entering the stationary phase with intermittent diauxic lags (labeled with arrows) (Fig. 3C). Thus, CgtA GTPase is necessary to support cellular growth when subjected to nutritional stress. Thus, the reduced growth rate in the *cgtA*-depleted cells appears to correlate well with the subcellular CgtA concentrations.

The doubling time of *ΔcgtA::kan^r^* (168 min) was approximately three times that of Wt (59.58 min) in M9 minimal media. The growth rate constant of Wt is 0.011 min^−1^, and that of *ΔcgtA::kan^r^* is 0.004 min^−1^.

Furthermore, the viability of *ΔcgtA::kan^r^* was severely affected. The log CFU/mL was estimated for Wt (~3.77 × 10^11^) and *ΔcgtA::kan^r^* (~360) (Fig. 3D). Therefore, as the nutrients decline in the media, the cells lose their viability. Hence, *cgtA* is critical in supporting the viability of V. cholerae during nutritional stress.

### Depleting full-length CgtA triggers starvation-induced persistence.

The *ΔcgtA::kan^r^* and the Wt cells growing in M9 minimal media (supplemented with glucose and casamino acids) were subjected to an extended incubation period at 37°C for 38 h without arabinose supplementation. The nutritional stress aggravates with the gradual depletion of the available carbon resources in the minimal growth medium. At the end of the incubation period, the cultures were serially diluted and plated on LB agar media to check for the resuscitation of persister cells.

Under the CgtA depletion condition, there was a steep reduction in the viable cells. After prolonged incubation (until 24 h), microcolonies of colonies of V. cholerae started emerging for the CgtA-depleted strain (Fig. 3E).

### The C-terminal domain of Gram-negative pathogens is evolutionarily distinct from Gram-positive pathogens.

**FIG 4:**
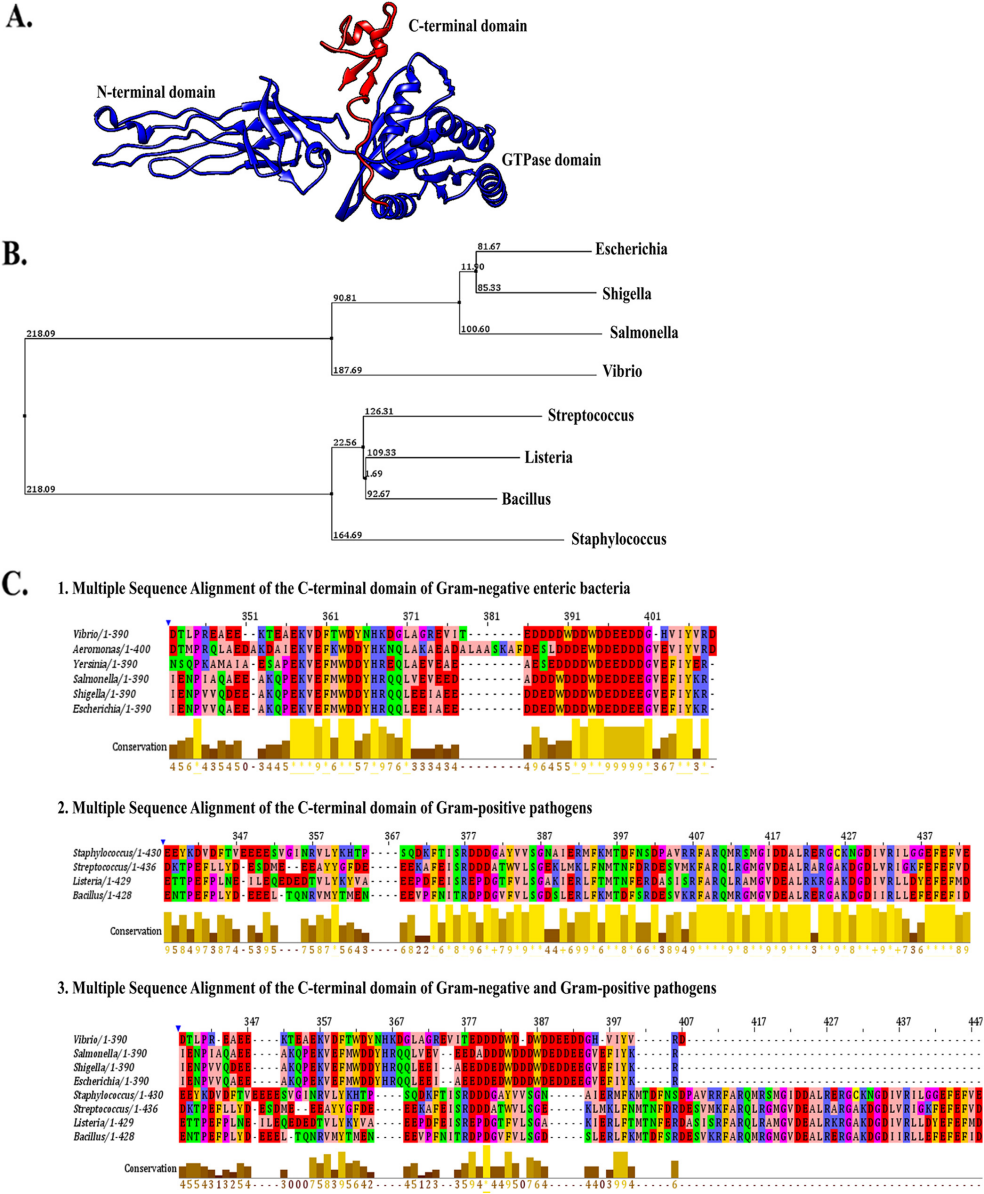
(A) Homology model of the full-length CgtA of Vibrio cholerae. (B) Phylogenetic analysis of the C-terminal domain of CgtA in both Gram-positive and Gram-negative enteric pathogens. (C) Multiple sequence alignment (MSA) of the C-terminal domain of CgtA in Gram-negative enteric pathogens (1), Gram-positive pathogens (2), and comparison between Gram-negative and Gram-positive pathogens (3).

CgtA is structurally unique from other GTPases due to its glycine-rich “Obg fold” with finger-like loops in the N-terminal domain. The highly flexible N-terminal domain acts as a shaft to anchor the 50S ribosome. The CTD length of CgtA varies widely (from 8 to 150 amino acids long) among bacterial species. How the CTD of CgtA functions *in vivo* is not well understood. Moreover, the CTD of CgtA is intrinsically disordered. The homology model of the full-length CgtA of Vibrio cholerae is depicted in Fig. 4A. UCSF Chimera version 1.15 (https://www.cgl.ucsf.edu/chimera/) was used to generate the structural model.

The phylogenetic analysis shows that the CTD of CgtA Gram-positive and Gram-negative pathogens evolved from a common ancestor and formed a monophyletic group. The CTD of Gram-negative pathogens is more closely related, and the same is true for Gram-positive pathogens (Fig. 4B).

Multiple-sequence alignment (MSA) was performed for three categories (Fig. 4C). In the first category (i), amino acid sequences of the CTD of CgtA from the Gram-negative enteric pathogens were considered.

In the second category (i), amino acid sequences of the CTD of CgtA from the Gram-positive pathogens were considered. In the third category (iii), amino acid sequences of the CTD from both categories A and B were combined and examined. In categories A and B, a high level of conservation profile of amino acids of the CTD was observed in both the Gram-negative and Gram-positive pathogens. In contrast, the conservation profile of amino acids was highly reduced in category C, implying that the CTD found in Gram-negative bacteria significantly varies in amino acid sequence and length from the Gram-positive bacteria.

### The composition of growth media impacts the growth characteristics of CgtA(ΔCTD).

**FIG 5:**
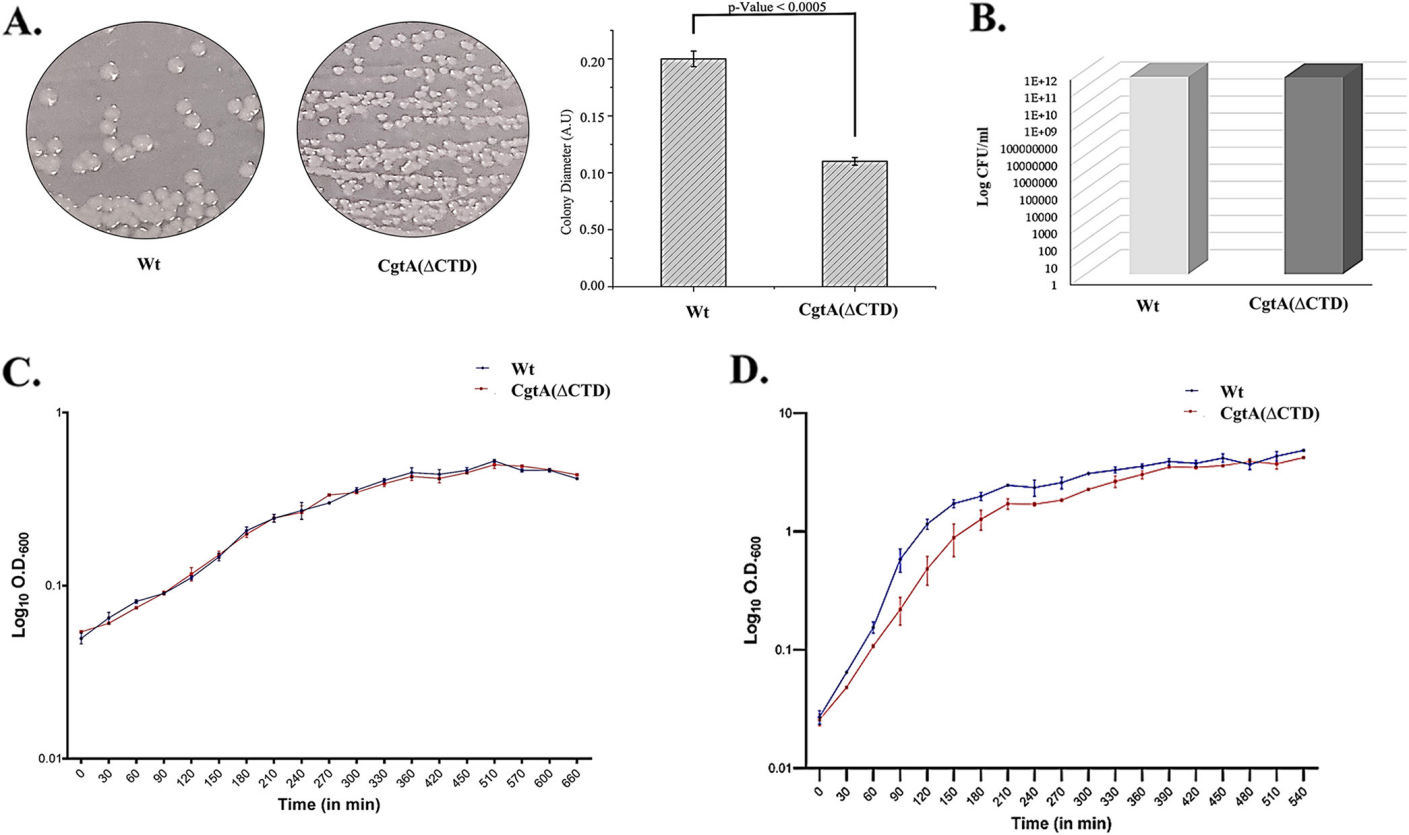
(A) Small-colony morphology of V. cholerae due to the truncation of the C-terminal domain of CgtA and quantification of the colony diameter. (B) Estimation of log CFU/mL Wt versus CgtA(ΔCTD) V. cholerae. (C) V. cholerae with CgtA(ΔCTD) M9 media exhibits the same growth pattern as Wt. (D) V. cholerae with CgtA(ΔCTD) grown in LB media grows at a slower rate than Wt. All the experiments were done in replicate, and the error bars are the standard error of the mean.

Robust growth of bacteria was observed for Wt. Small-colony variants of CgtA(*Δ*CTD) were observed compared to the Wt strain. The resultant small-colony morphology due to the truncation of the C-terminal domain of CgtA implies a growth defect (Fig. 5A). The viability of V. cholerae cells bearing a deletion of the C-terminal domain of CgtA was not affected compared to Wt. The log CFU/mL estimated was equivalent to the Wt cells (Fig. 5B).

The growth curve experiments for the Wt and CgtA(ΔCTD) strains were performed in nutrient-rich LB medium and M9 minimal medium supplemented with glucose and Casamino Acids. In M9 minimal media, the growth pattern of Wt and CgtA(ΔCTD) followed a similar pattern, as evident from the growth curve (Fig. 5C). An interesting observation was made in the LB media when the growth curve experiment was performed. The CgtA(ΔCTD) strain showed a retarded growth pattern in LB media compared to its Wt counterpart (Fig. 5D). The doubling time of the CgtA(ΔCTD) was estimated to be 32 min in LB media. The doubling time was approximately 19 min for the Wt strain. The doubling time of Wt V. cholerae grown in LB at 37°C is reported to be between 16 and 20 min (18). Hence, the nature of the growth media influences the growth pattern of CgtA(ΔCTD).

### Depleting full-length CgtA from cells leads to a distinguished morphological appearance.

The morphologies of Wt, full-length CgtA-depleted cells, and CgtA(ΔCTD) cells were studied by growing in nutrient-rich LB and nutrient-limiting M9 minimal media.

**FIG 6:**
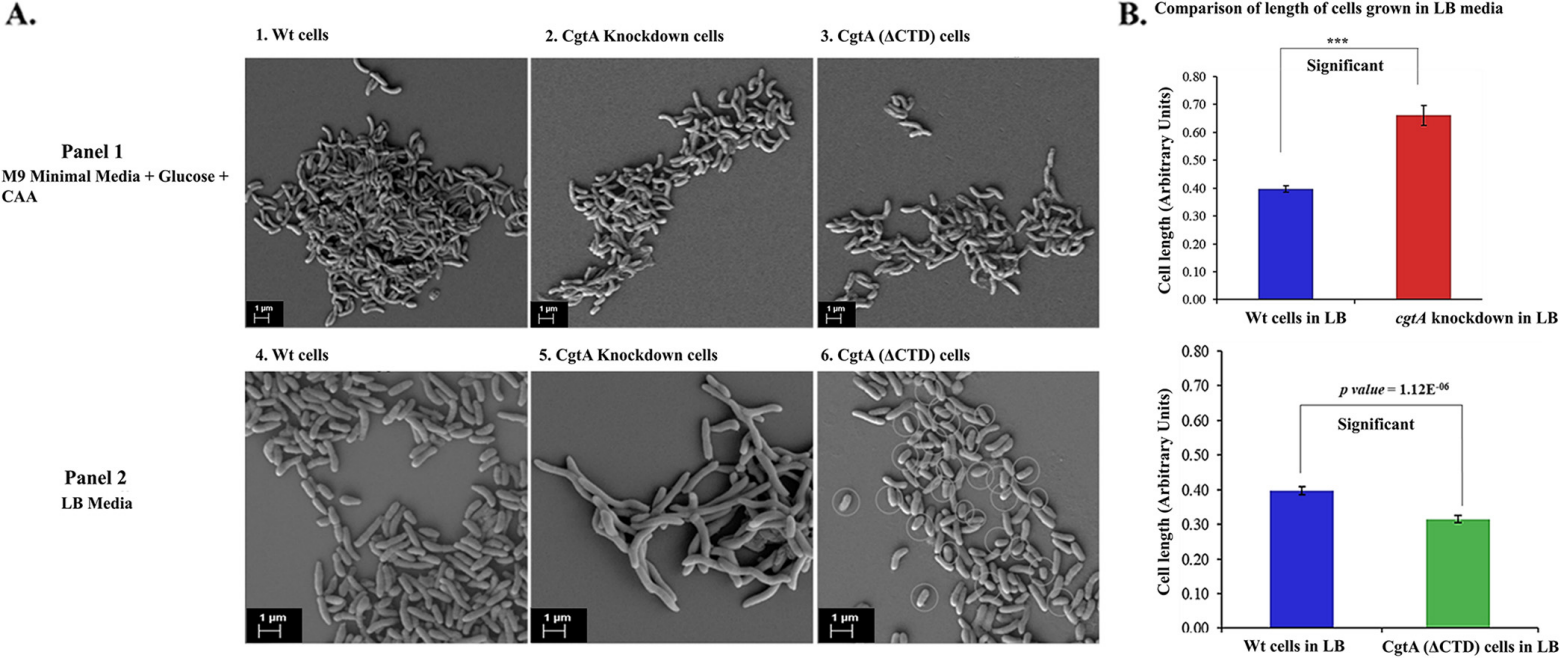
(A) Scanning electron microscopy imaging. (A, panel 1) Wt, full-length *cgtA* knockdown, CgtA(ΔCTD) cells grown in M9 media. (magnification, 5.00 KX). (A, panel 2) Wt, full-length *cgtA* knockdown, CgtA(ΔCTD) cells grown in LB media (magnification, ~10.00 KX). (B) The cell lengths for all three strains grown in LB were quantified and statistically validated using a two-tailed *t* test.

In the nutrient-limited M9 minimal media, there was no remarkable difference between the Wt and *cgtA*-depleted cells of V. cholerae (Fig. 6A, panels 1 and 2). The cells of both strains appeared indistinguishable. Also, no noticeable difference was observed for CgtA(ΔCTD) cells (Fig. 6A, panel 3).

When the CgtA-depleted V. cholerae was cultured in nutrient-rich LB media, a distinct morphological pattern was observed under the scanning electron microscope (SEM). The CgtA-depleted cells appeared highly elongated, stressed, and filamentous, contrary to the normal morphology of the Wt grown until the mid-exponential phase (Fig. 6A, panels 4 and 5). Interestingly, a distinct population of CgtA(ΔCTD) cells belonging was smaller in length than the Wt cells (Fig. 6A, panel 6). The cell lengths for all three strains grown in LB were quantified and statistically validated using a two-tailed *t* test (Fig. 6B). The observation is commensurate with the delayed growth in LB media for CgtA(ΔCTD) cells.

### CgtA regulates chemotaxis in Vibrio cholerae.

**FIG 7:**
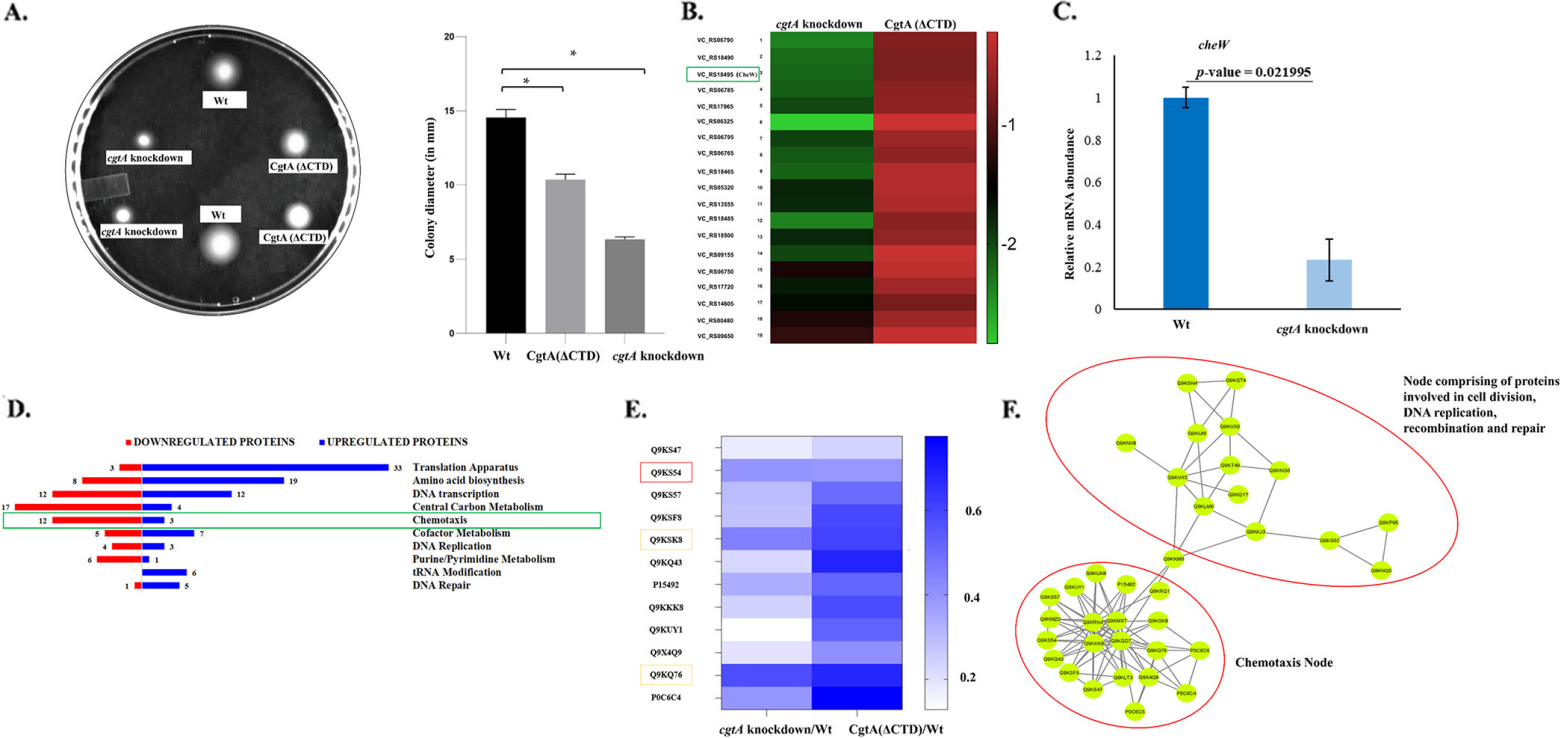
(A) Analysis of motile characteristics by soft agar motility assay of Wt, full-length CgtA depletion, and CgtA(ΔCTD) V. cholerae. (B) Heatmap showing the chemotaxis genes significantly downregulated in full-length *cgtA* knockdown V. cholerae. (C) qRT-PCR of the *cheW* gene involved in chemotactic motility and which works as a scaffolding protein in chemotactic arrays. (D) Chemotaxis among the top 10 enriched ontology groups under the CgtA-depleted condition of V. cholerae. (E) Involvement of the C-terminal domain of CgtA in chemotaxis and flagellar motility. (F) Interaction between the node of chemotaxis proteins and the node of proteins related to DNA replication and chromosome partitioning under the full-length CgtA-depleted condition.

The role of CgtA in impacting the motile behavior of V. cholerae is crucial to understanding its environmental adaptations. To understand the effect of full-length CgtA on the motile behavior of V. cholerae, both the Wt and Δ*cgtA*::*kan^r^* strains were tested for their motility using soft agar motility assay. It was observed that the motility of V. cholerae is severely affected when *cgtA* is knocked down from the V. cholerae genome (Fig. 7A).

It was observed that the extent of decrement of motility was less in the case of CgtA(ΔCTD) than the full-length *cgtA* knockdown strain (Fig. 7A). Thus, in V. cholerae, full-length CgtA plays a significant role in controlling motility. In contrast, the C-terminal domain of CgtA also controls motility, but the influence is less than full-length CgtA. It can be concluded that apart from the C-terminal domain, the N-terminal and the GTPase domains are critical in influencing the motile characteristic of V. cholerae.

The chemosensory system of V. cholerae is exquisitely complex and categorized into three types, out of which only one (cluster II/F6 system) is reported to affect chemotactic motility under any tested conditions (19). The functions of the other two types are not yet discovered. Over 50 chemosensory proteins are expressed from all three categories.

In search of the underlying cause of the reduced motility in the *cgtA*-depleted strain, genome-wide high-throughput studies were carried out to search for the change of expression of the motility genes in mRNA and proteome levels. RNA-Seq studies (Fig. 7B) revealed that many genes and proteins related to the chemotaxis were downregulated in the CgtA-depleted V. cholerae strain. The CheW protein is involved in chemotactic motility and works as a scaffolding protein in chemotactic arrays. The mRNA abundance of *cheW* is low under the CgtA-depleted condition compared to Wt (Fig. 7C).

Our results from the proteomics study also revealed that a set of 20 proteins with biological processes of flagellar motility and chemotaxis was among the top 10 enriched ontology groups under the CgtA-depleted condition of V. cholerae (Fig. 7D). The chemotaxis sensor kinase proteins, namely, CheA, CheY, and CheV, were decreased, and many methyl-accepting chemotaxis proteins (MCPs) were differentially regulated (Table S1A). The magnitude of downregulation of those 12 proteins is visualized with the help of a heatmap. It can be inferred that the proteins involved in the chemotactic motility function of V. cholerae are more downregulated when the cells are completely depleted of full-length CgtA. A total of 12 chemotaxis and flagellar motility proteins were significantly downregulated under both conditions, full-length *cgtA* knockdown and CgtA(ΔCTD) deletion (Table S2). Thus, the results obtained from the soft agar motility assay can be correlated with the heatmaps (Fig. 7B and Fig. 7E). Thus, the decreasing order of motility is as follows: wild-type cells, ΔCTD *cgtA::kan^r^*, and *cgtA::kan^r^* V. cholerae strains.

To identify the groups of proteins that interact with the cluster of chemotaxis proteins, we visualized the protein interactions by the STRING protein-protein interaction database server. According to the predicted protein-protein interactions by the STRING database, it is found that the node of chemotaxis proteins is interacting with the node of proteins related to DNA replication, recombination, and cell division (Tables S1B and C) under the Δ *cgtA::kan^r^* condition of V. cholerae (Fig. 7F). The following noteworthy proteins mentioned here are involved in chromosome partitioning: ParB family protein (VC_A1114), ParA family protein (VC_2773), and a chromosome segregation ATPase (VC_A1075).

### Implementation of proteomics to understand the intracellular role of the C-terminal domain of CgtA.

**FIG 8:**
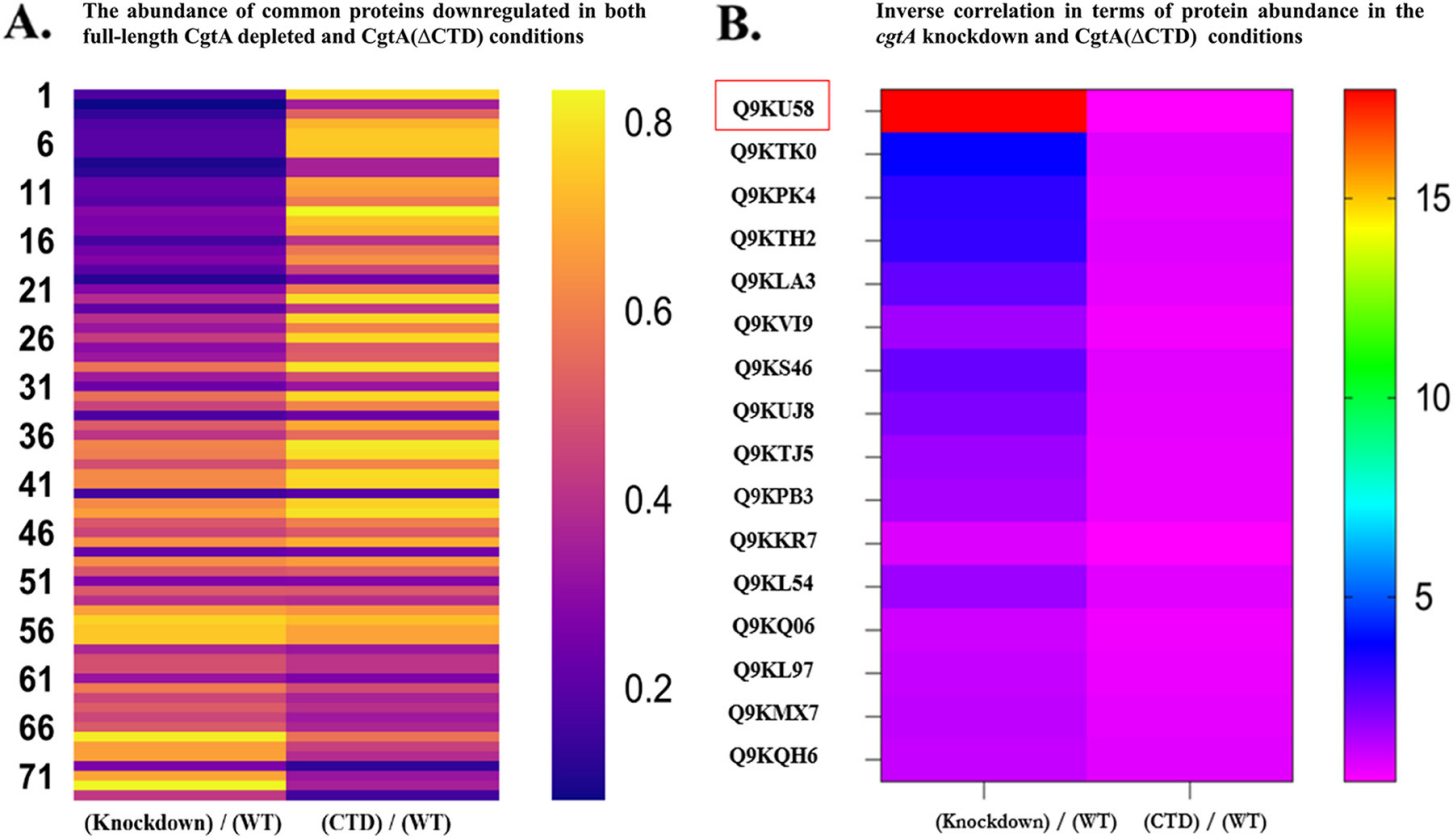
(A) Biological functions commonly downregulated under the full-length CgtA deletion and CgtA(ΔCTD) conditions. (B) Proteins significantly downregulated under the CgtA(ΔCTD) condition but significantly upregulated or unaltered under the CgtA-depleted condition.

The results from proteomics studies also identified the biological functions commonly downregulated under the full-length CgtA depletion and CgtA(ΔCTD) conditions. Based on the ANOVA test and fold change cutoff, proteins significantly downregulated under both full-length CgtA deletion and CgtA(ΔCTD) conditions are depicted using a heatmap (Fig. 8A). The abundance ratio of proteins ranged from 0.2 to 0.83 (fold change of 0.83 is the cutoff for downregulation). Out of 311 significantly altered proteins under the full-length CgtA depletion condition and 240 proteins significantly altered under the CgtA(ΔCTD) condition, a total of 76 proteins were common to both groups that were downregulated (Table S3). Even though all 76 proteins are downregulated under both conditions, the degree of downregulation varies. A heatmap was used to visualize the extent of downregulation under both conditions for each protein enlisted in the table of this section. Similar expression of the common downregulated proteins is also evident by the color scale that signifies the fold change level. Chemotaxis, transcription, and amino acid metabolism are the three most downregulated biological processes due to full-length CgtA depletion and CgtA(ΔCTD) deletion in V. cholerae.

Furthermore, proteins significantly downregulated under the CgtA(ΔCTD) condition but significantly upregulated or unaltered under the CgtA-depleted condition are also identified in the proteomics study. Sixteen proteins were sorted based on significant downregulation when the C-terminal domain of CgtA was truncated. In contrast, those 16 proteins were significantly upregulated or remained unaltered when the full gene of *cgtA* was knocked down from the genome of V. cholerae. This inverse correlation of the altered proteins is visualized using a heatmap (Fig. 8B) and listed in Table S4. An uncharacterized protein, Q9KU58, is anomalously upregulated during full-length *cgtA* deletion (fold change of 17.722), whereas under the CgtA(ΔCTD) condition, the fold change is only 0.367.

### Abnormal protein translation takes place upon full-length CgtA depletion.

**FIG 9:**
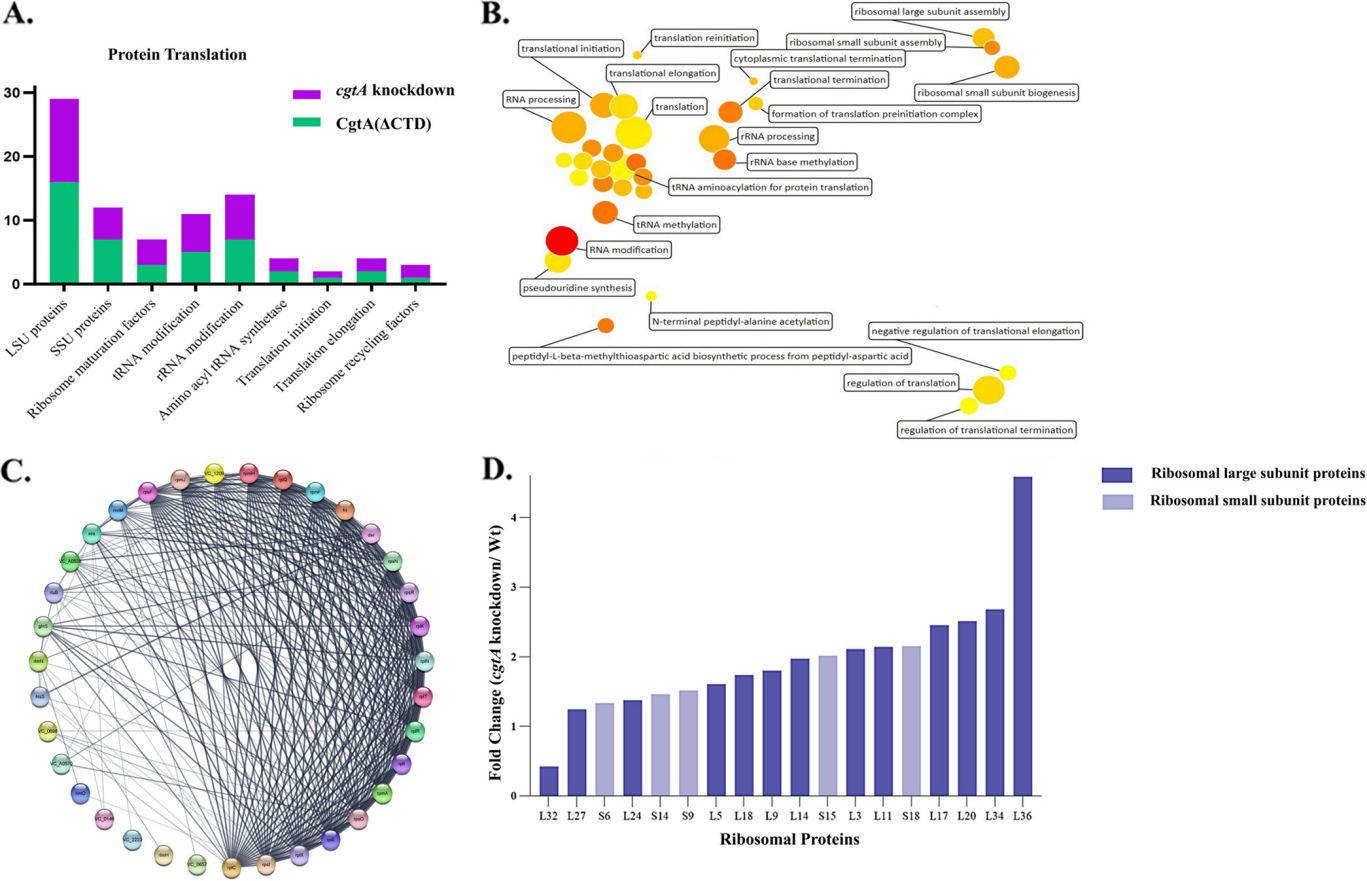
(A) Stacked bar chart showing the distribution of proteins involved in ribosome biogenesis and protein translation affected under the full-length CgtA depletion and CgtA(ΔCTD) conditions. (B) Visualization of the altered translational proteins with the help of a bubble plot involved in the process of protein-translation apparatus under both full-length CgtA depletion and CgtA(ΔCTD) conditions. (C) Network of the translational proteins downregulated under the full-length CgtA depletion condition. (D) Upregulation of 50S and 30S ribosomal subunit proteins under the full-length CgtA depletion condition.

The proteomics study results indicate a high perturbation in the cell arising from the high demand for ribosomal protein biosynthesis (Fig. 9A).

With the help of a web-based tool, Revigo, all the altered translational proteins are visualized with the help of a bubble plot (http://revigo.irb.hr/) (Fig. 9B). The bubble color indicates the input *P* value, and the bubble size indicates the frequency of the GO term. The details of the significantly altered proteins of the protein translation apparatus of V. cholerae are listed (Table S5). The translational proteins downregulated under the CgtA depletion condition form a network among themselves (Fig. 9C).

Seventeen 50S and 30S ribosomal subunit proteins are significantly upregulated under the *cgtA* knockdown condition (Fig. 9D). One protein of the large subunit, L32, was significantly downregulated. Among the large subunit (LSU) ribosomal proteins that are significantly upregulated are L9, L5, L36, L34, L27, L24, L20, L18, L17, L14, and L11. The L36 protein was found to be 4.5-fold abundant under CgtA-depleted conditions. L3, L11, L17, L20, and L34 proteins were greater than 2-fold but less than 3-fold in abundance. L5, L9, L24, L27, L14, and L18 proteins were greater than 1-fold but less than 3-fold in abundance. Among the small subunit (SSU), significantly upregulated proteins are S6, S9, S14, S15, and S18. S6, S9, and S14 proteins vary between 1- to 2-fold abundance. At the same time, S15 and S18 are approximately 2-fold abundant.

Several rRNA- and ribosomal protein-modifying enzymes are highly abundant in the cells devoid of the CgtA protein. Among these enzymes are the RluB, RlmN, and RlmH involved in modifying the 23S rRNA of the LSU. RluB is involved in the pseudouridine synthesis from uracil-2605 in 23S rRNA. RlmH methylates the pseudouridine at position 1915 in the 23S rRNA. RlmN plays a crucial role in the proofreading step at the peptidyl transferase center by specifically methylating position 2 of adenine 2503 in the 23S rRNA. RsmD is the only significantly upregulated 16S rRNA-modifying enzyme that methylates the guanine in position 966. RimI and RimO are the two significantly upregulated enzymes involved in SSU ribosomal protein modification. RimI modifies the S18 protein by N-terminal alanine acetylation. RimO methylthiolates an aspartic acid residue of the S12 protein.

Some accessory translation factors involved in protein translation are significant. The initiation factor, Sui1, is significantly upregulated (~1.8-fold abundant). Furthermore, elongation factor P-like protein (a close homolog of EF-P) is significantly downregulated. An energy-dependent translational throttle protein, EttA, sensitive to the ATP/ADP ratio, modulates the progression of the 70S ribosomal initiation complex into the elongation cycle, and is significantly upregulated. The peptide chain release factor 3 (RF3), involved in forming ribosomal termination complexes with a strong preference for UGA strong codons, is significantly upregulated. The ribosome-recycling protein, responsible for increasing the translation efficiency by recycling the ribosomes, is significantly downregulated.

Several ribosome maturation factors, namely, Der, Era, RimM, and RbgA, are also significantly upregulated. RimM and Der were more abundant (greater than 3-fold abundance) than Era and RbgA. The Era GTPase is involved in the 16S rRNA processing and 30S ribosomal subunit biogenesis. The RbgA GTPase is involved in the late-step assembly of the 50S ribosomal subunit. RimM is involved in the final step assembly of the 30S ribosomal subunit assembly. Der GTPase is essential for the late steps of ribosome biogenesis by stabilizing the 50S ribosomal subunit to facilitate its assembly into the 70S ribosome. CgtA GTPase is involved in the late steps of LSU assembly (10). It could be possible that, as a last resort for suppressing the ribosome assembly defects, alternative ribosome maturation factors involved in 50S ribosomal subunit biogenesis, like RbgA and Der GTPases, are overexpressed. At the same time, 30S ribosomal subunit biogenesis proteins Era GTPase and RimM are also overexpressed. Additionally, two aminoacyl tRNA synthetases, glutamine tRNA ligase and histidine tRNA ligase, were significantly upregulated. Moreover, many enzymes involved in tRNA modification were upregulated manifold.

### Correlation between proteomics and transcriptomics data under Wt versus full-length CgtA depletion conditions.

**FIG 10:**
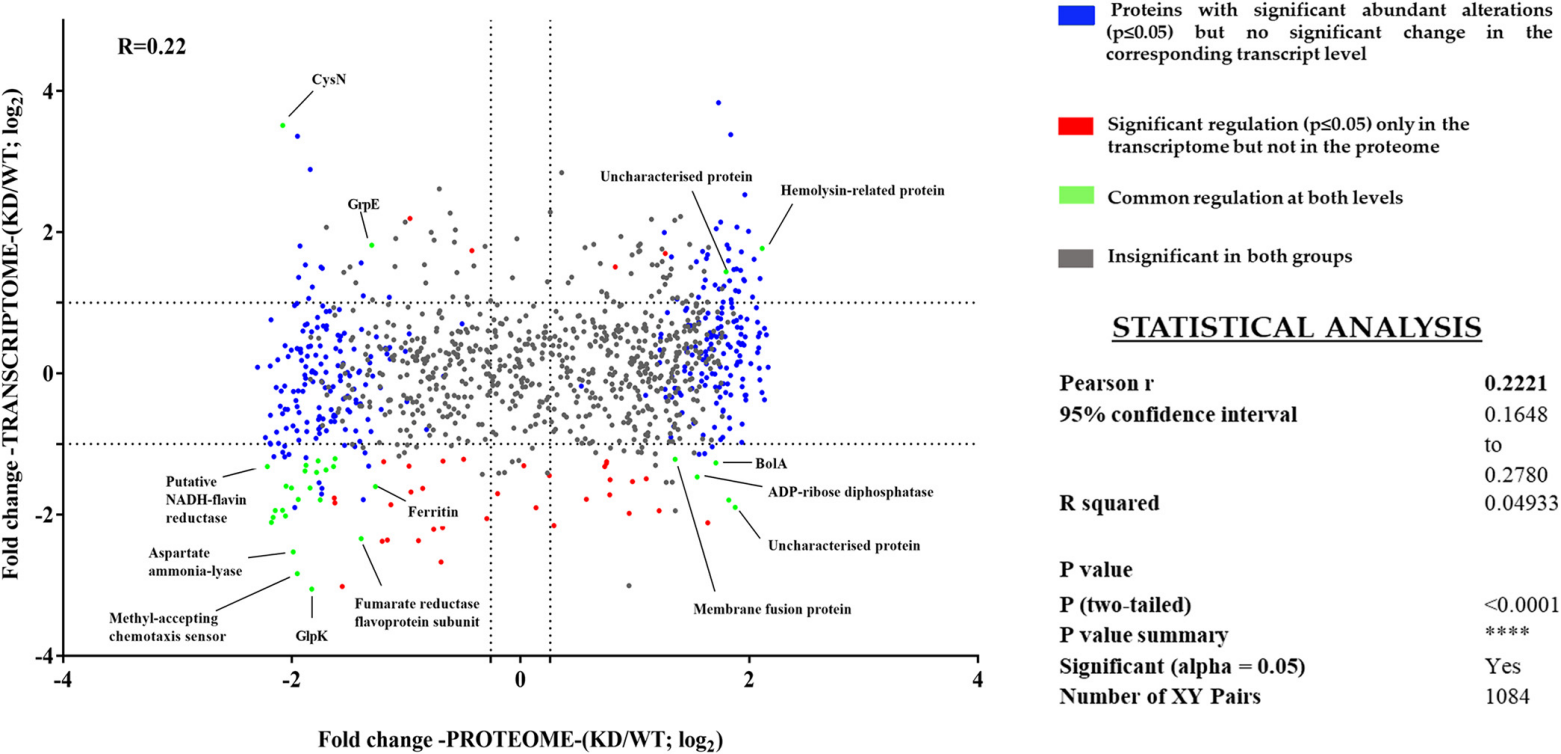
Correlation plot to understand the integrated proteomics and transcriptomics data. The log_2_ fold change of the altered transcriptome is plotted with the log_2_ fold change of the altered proteome for the *cgtA* knockdown condition. A total of 1,084 XY pairs are plotted. Pearson’s correlation coefficient (*R*) was calculated to determine how the two data sets are connected. The proteins are clustered into four different groups based on the significant alterations at both transcriptome levels. The protein clusters are color coded. The expression of genes significant at both the transcriptome and proteome levels are green in color.

Both the proteomics and the transcriptomics data were integrated and analyzed with the help of a correlation plot (Fig. 10). In this plot, the log_2_ fold change of the altered transcriptome is plotted with the log_2_ fold change of the altered proteome for the *cgtA* knockdown condition. The cutoff is denoted as dotted lines on the *x* and the *y* axes for the fold changes. Student’s *t* tests were performed for transcriptomics and proteomics data, and the proteins with significant *P* values (≤0.05) were sorted. While plotting, the proteins were clustered into four groups based on the significant alterations at both transcriptome levels. The protein clusters were color coded. The expression of genes significant at both the transcriptome and proteome levels are green in color. Some of them are labeled in the correlation plot. A total of 1,084 XY pairs are plotted. Pearson's correlation coefficient (*R*) was calculated to determine how the two data sets are connected. A positive correlation was found where *R* equals 0.22 (0 < 0.22 < 1). Significant differential regulation of 30 genes at both the mRNA and protein expression levels was common (Table S6).

### The temporal expression pattern of *cgtA* is influenced by the characteristic of growth media.

The growth pattern of Wt V. cholerae due to nutritional stress upon shifting the exponentially growing bacterial culture from nutrient-rich LB media to M9 minimal media was studied. It was observed that the cells entered a very short lag phase upon nutritional downshift to adapt to the nutrient-poor environment, followed by resuming growth, reentering into the logarithmic phase, and finally again entering into the stationary phase (Fig. S4).

**FIG 11:**
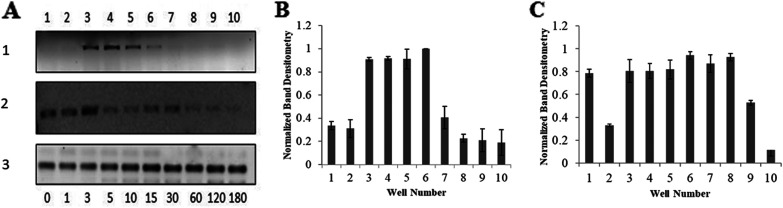
Expression pattern of CgtA. (A) Semiquantitative RT-PCR of *cgtA* mRNA from 1 μg of total RNA isolated from V. cholerae cells collected at different time intervals. (A1) Expression of *cgtA* in control cells growing in LB media. (A2) Expression level of *cgtA* after shifting to M9 minimal media. (A3) Expression of the IF2 gene as control. (B and C) Histograms depicting the band intensity corresponding to panel A1 (B) and panel A2 (C), respectively.

RNA was extracted from the bacterial samples at different time points during different growth phases initially in LB and post-nutritional downshift in M9 minimal media. In nutrient-rich LB media, the expression of *cgtA* mRNA first increases in the logarithmic phase and then gradually decreases in the stationary phase (Fig. 11A and B). Similar findings were also reported earlier in E. coli stating that GTP-bound Obg proteins are involved in cell growth and are inactivated during the stationary phase (6).

In contrast, after nutritional downshift into the minimal media, the mRNA expression pattern of *cgtA* is sustained for a longer period than LB media (Fig. 11A and C). The detailed statistical analysis of the semiquantitative PCR data is provided in Table S9. The prolonged *cgtA* expression corresponds to the slow growth in M9 minimal media. Furthermore, similar to LB media, the expression of *cgtA* mRNA in M9 minimal media also decreases gradually while entering the stationary phase. Thus, the nutrient content of the growth media influences the temporal expression of *cgtA* of V. cholerae.

## DISCUSSION

CgtA is an essential GTPase. To withstand various environmental stresses, bacteria elicit several stress responses for survival. The nutritional stress response is prevalent in bacteria as nutrients decline in the growth media. CgtA represses the onset of stringent response in V. cholerae (5). The role of CgtA is limited only to maintaining steady-state levels of (p)ppGpp but is not responsible for (p)ppGpp accumulation during amino acid starvation (2). In E. coli, it is reported that CgtA primarily associates with the 50S ribosomes and interacts with SpoT ppGpp hydrolase/synthetase, a stringent response protein (13). In V. cholerae, depletion of CgtA leads to global transcriptional changes accordant with the stringent response in LB media (nutrient rich) (5). Moreover, increased ppGpp concentration was reported in *cgtA*-depleted V. cholerae cells (5).

The hypothesis presented through this work is that under nutrient-limiting conditions (M9 minimal media), V. cholerae depleted of full-length CgtA would undergo stress and display an aggravated pattern of altered transcriptome and proteome that takes part in nutrient stress response, including the general stress-responsive genes. Searching those genes and proteins by genome-wide transcriptomics and proteomics approaches unfolded various novel findings.

Various tRNA genes were downregulated in cells deprived of full-length CgtA grown in M9 minimal media. This observation generates model (Fig. S8). It suggests how the stressful conditions arising during nutrient-poor conditions lead to the downregulation of tRNA (20) by a reduced rate of transcription and degradation by RNase E and polyadenylation, eventually leading to the cessation of translation and subsequent cellular growth. Thus, we add an additional factor, i.e., full-length CgtA depletion, that aggravates the intracellular stress manifolds (Fig. S8).

The C-terminal domain (CTD) length of CgtA varies widely (from 8 to 150 amino acids long) among bacterial species. How the CTD of CgtA functions *in vivo* is not well understood. Moreover, the CTD of CgtA is intrinsically disordered. The contribution of the CTD of CgtA nucleotide binding and hydrolysis has been reported in *E. coli* (21). The deletion of the CTD displayed a 10-fold decrease in affinity for GTP but a small decrease in affinity for GDP (21). The decrease in affinity for GTP is due to the high GTP dissociation rate (21).

In contrast, CTD is not substantially involved in the GTP turnover (21). Thus, in E. coli, the CTD of CgtA is implicated in the binding of GTP with very little effect on GDP binding. A suggested mechanistic role of E. coli CgtA’s CTD is that it folds back on the GTPase domain in the GTP binding state, which is reflected in decreased affinity for GTP (21). Due to the lack of conservation of the CTD of CgtA among various organisms, it remains inconclusive whether such a role of CTD is general or specific. The CTD of the CgtA of V. cholerae is 57 amino acids long. Previously, it was reported from the *in vitro* studies that the GTPase activity of the CTD-deleted CgtA is elevated when coupled with the 50S ribosome than the Wt CgtA, implying a negative regulatory effect of the CTD on the overall GTPase activity in the 50S ribosome interacting state (22). In this current study, from the MSA and phylogenetic analysis, it can be concluded that the CTD of Gram-negative bacteria evolved separately from the Gram-positive bacteria, becoming more distinct over time. Thus, the functional roles of the CTD might also vary based on evolutionary diversification.

Comparative proteomic analyses of Wt versus CgtA knockdown and Wt versus CgtA(ΔCTD) revealed intriguing cellular aspects of metabolic fitness of V. cholerae. Even though there are many biological processes commonly downregulated under both conditions, the cause of the essentiality of full-length CgtA in a *cgtA* knockdown strain might be due to the more weakened metabolism due to the greater number of proteins involved in each of the biological functions or maybe even the more diverse number of critical cellular pathways affected. Additionally, the most probable reason for the inverse correlation of the proteins differentially regulated in Wt versus CgtA knockdown and Wt versus CgtA(ΔCTD) is that the expression of those proteins got affected when the CTD of CgtA was deleted exclusively, whereas upon full-length *cgtA* deletion, the extremely high upregulation of those proteins is due to the expression via an abnormal pathway.

The distinct morphological pattern of V. cholerae cells depleted of CgtA grown in LB could be an outcome of an imbalance between stringent response and a high growth rate in LB. Bacterial cells actively grow and replicate their DNA during the exponential-growth phase. The ongoing molecular and cellular processes are highly stressful when bacteria undergo rapid growth (nutrient-rich media). These processes include protein translation, rapid DNA replication, cell division, and cellular reorganization (15). It is reported that bacterial cells demonstrate uncoupling between cell division, DNA replication, and segregation every 20 to 40 min under optimal conditions. As chromosomal DNA is highly condensed and ~1,000 times the cellular length, the segregation of DNA requires a longer time (60 to 90 min) (15). Hence, the highly significant morphological differences of the *cgtA*-depleted strain under two different growth conditions (nutrient rich and nutrient poor) are because CgtA GTPase is essential to modulate the stringent response in nutrient-rich LB media proportionate to the high growth rate (23). In contrast, CgtA does not play any role in modulating the stringent response during growth in minimal media, which is nutrient limiting and hence does not support rapid growth. Hence, depleting the cells of CgtA and growing in minimal media does not affect cell division.

For a long time, it has been known that there is a link between nutritional stress and its manifestation in V. cholerae in the form of metabolically inactive cells known as “persister cells.” Persister cells are a group of stochastically formed low-energy cells due to the alterations in the energy-generating components.

Sporadic outbreaks of cholera have been reported when the environmental conditions of V. cholerae become favorable when the persister cells transition from the dormant state to the proliferative state. The nutritional stress aggravates due to the depletion of the available carbon resources in the growth media. The resuscitation of deeply starved V. cholerae cells occurred when regrown on nutritionally enriched media. Thus, depletion of CgtA from the V. cholerae cells elicits an intense starvation-induced stress response that results in the formation of persister cells. When the culture conditions become favorable (availability of nutrient-rich medium), the growth of the persister cells resumes. Hence, CgtA supports the long-term survival of starvation-induced V. cholerae cells.

In contrast, there was no evidence of microcolonies (persister cells) in CgtA(ΔCTD) cells, unlike under full-length CgtA-depleted condition. Hence, the role of the CTD of CgtA is not to trigger the nutritional stress response in V. cholerae.

From the comparative growth studies in LB versus M9 minimal media, it is suggested that the role of the CTD of CgtA in V. cholerae is to herald the nutrient levels of the growth media to the other two domains, the N-terminal domain and the GTPase domain, in order to suppress the onset of stringent response by regulating the steady-state (p)ppGpp levels.

In addition, depleting full-length CgtA and truncating the CTD of CgtA affected the motility of V. cholerae. Motility is a dynamic feature of various microbes. V. cholerae is monotrichous, i.e., it possesses a single polar flagellum that helps it swim. Motility is dependent on environmental stimuli, interactions with other microorganisms, and even pathogenic character. V. cholerae exhibits a back-and-forth motion by reversing the direction of the flagellar motor. The role of CgtA in impacting the motile behavior of V. cholerae is crucial to understanding the environmental adaptations of V. cholerae. The proteins related to chemotaxis affected due to full-length CgtA depletion are summarized as follows. Methyl-accepting chemotaxis proteins (MCPs) are the largest class of chemotaxis proteins that showed significant downregulation (a few MCPs were upregulated too). MCPs, the most common receptors of bacteria, are arranged in dimers forming hexagonal arrays in either the cytoplasm or cytoplasmic membrane (24). MCPs are involved in the chemotactic signal transduction from the environment to the downstream cytoplasmic chemotaxis proteins (24). Hence, the signal recognition by MCPs in cells deprived of CgtA is affected, resulting in defective modulation of its motile behavior.

Another significantly upregulated protein seen during CgtA depletion is CheC (VC_A0189). CheC acts as an inhibitor of methylation of the MCPs but does not interfere with CheB (methyltransferase). The mechanism of the CheC function is unknown (25). Hence, in the absence of CgtA, higher levels of CheC impede the methylation of the signaling domains of MCPs.

The MCPs of V. cholerae exist in clusters and are known to localize to the polar regions of the cell (26). Several reports suggest a linkage between cell division and chemotaxis in E. coli and V. cholerae. It is known that the core chemotaxis unit, also known as the ternary core-signaling complex, comprises CheA, CheW, and MCPs (27). Multiple core units are arranged into a superlattice structure known as the chemosensory array (28). The chemosensory arrays are highly electron dense and parallel to the inner membrane. Multiple chemosensory arrays are further arranged to form highly ordered macromolecular complexes. The highly ordered chemosensory signaling arrays mediate chemotactic cooperativity, signal transduction, and amplification (29). The ParA-like ATPases (ParA1, ParC, and FlhG) are anchored to the cell poles via a transmembrane protein, HubP, ensuring the polar localization of the chemotactic machinery, flagellum, and chromosome origin (30). Another critical factor determining the positioning of the chemosensory arrays of V. cholerae at the cell poles is the AIF domain of the ParP protein, the partner protein of ParC (31).

It is reported that the cells deficient in ParC, ParP, or both have reduced chemotactic motility (26). The bipolar pattern of ParC and ParP protein localization is similar to the chemosensory arrays. As the cell cycle progresses, there is consecutive recruitment of HubP, followed by ParA1, to the new cell pole. Once the chromosome segregation is initiated, ParA1 recruits ParB1 to the new cell pole. ParC is then redistributed to the new pole from the old pole, followed by the recruitment of ParP. ParP then aids in forming a new chemosensory array at the other end of the pole after the completion of cell division (31). Several cell division proteins are downregulated under the CgtA-depleted condition. According to the predicted protein-protein interactions by the STRING database, it was found that the node of chemotaxis proteins is interacting with the node of proteins related to DNA replication and chromosome partitioning when *cgtA* is knocked out. The noteworthy proteins to be mentioned here are involved in chromosome partitioning and include ParB family protein (VC_A1114), ParA family protein (VC_2773), and a chromosome segregation ATPase (VC_A1075). In the CgtA depletion background, it is tempting to speculate that the lowered expression of cell division proteins interferes with the proper arrangement of chemosensory arrays. It henceforth leads to disrupted chemotactic signaling followed by reduced swarming motility. The magnitude of downregulation of those 12 proteins is visualized with the help of a heatmap. It can be inferred that the proteins involved in the chemotactic motility function of V. cholerae are more downregulated when the cells are completely depleted of full-length CgtA. Thus, the heatmap can explain the results obtained from the soft agar motility assay. Thus, the decreasing order of motility is as follows: Wt, Wt (ΔCTD), and *cgtA* knockdown V. cholerae strains. Hence, it can be concluded that the CTD of CgtA also controls motility, but the influence is less than full-length CgtA. Also, the N-terminal domain and the GTPase domains are critical in influencing the motile characteristic of V. cholerae.

The proteomics study also revealed that the protein translation process was abrupt when both full-length CgtA and the CTD of CgtA were deleted. However, under the full-length CgtA depletion condition, the extent of dysregulation was maximum. The translational machinery of prokaryotes is multicomponent. Protein translation is an energy-dependent and complex process involving multiple steps. It encompasses the biosynthesis of 50S and 30S ribosomal subunits and the precision assembly of the subunits to form the 70S ribosome. Any dysregulation of ribosome biogenesis can result in several deleterious effects in the cell.

This observation shows that Era, Der, RimM, and RbgA act in unison to compensate for the loss of CgtA. Hence, overexpression of one ribosome factor potentiates the compensation of the loss of another ribosomal factor within the cell (32). Even though CgtA is implicated in late-stage 50S ribosomal subunit assembly, the ribosome maturation factors compensating for its loss are involved in both 50S and 30S ribosomal subunit assembly. The observation, as mentioned earlier, can interpret functional interaction between the ribosome assembly factors. Similar observations were also reported when RbgA GTPase in B. subtilis was depleted (33). Many translation factors and ribosomal proteins were upregulated upon RbgA depletion. The cause for this upregulation of proteins was considered abnormal in connection with its role in ribosome biogenesis (33).

RelA and SpoT are the two critical stringent response proteins in bacteria. Guanosine tetraphosphate (ppGpp) and guanosine pentaphosphate (pppGpp) are alarmones that activate the stringent response, the ubiquitous bacterial stress signaling system in response to nutritional and environmental stresses (34). During amino acid starvation, RelA synthesizes the alarmone (p)ppGpp (2). In contrast, SpoT is predominantly responsible for (p)ppGpp hydrolysis and sometimes for synthesizing (p)ppGpp under various cellular stresses (2). RelA is associated with translating ribosomes, SpoT is associated with pre-50S particles, and CgtA is associated with the late stages of ribosome assembly (2). Hence, the association of SpoT and CgtA with ribosomes are mutually independent (2).

Initially, it was reported that the essentiality of CgtA lies only in the *relA*^+^ background and not in the *relA*^−^ background (5). Later on, it was demonstrated that *cgtA* is an essential gene in V. cholerae, even with a Δ*relA* genetic background (35). Thus, it implies that even though CgtA plays an important role in regulating the stringent response in V. cholerae, its essentiality is maintained irrespective of the elicitation of the stringent response. The transcriptome profile of CgtA-depleted V. cholerae cultured in LB was similar to that of the cells in which stringent response is elicited (5). From our results, it is clear that even in M9 minimal media, i.e., nutrient-limiting conditions, depleting CgtA triggers various abnormal metabolic activities, as evident from RNA-Seq and proteomics studies, despite no apparent changes in morphology being observed. Hence, it can be concluded that the growth rate of cells as influenced by the characteristic of growth media is the defining factor for the emergence of visible changes in phenotypic alterations under CgtA-depleted conditions. Also, mRNA and protein expression levels of RelA are not significantly altered for cells depleted of CgtA growing in minimal media supplemented with a low concentration of Casamino Acids (data not shown). Hence, even though the nutrient composition of M9 minimal media is significantly poorer than nutrient-rich LB, the elicitation of the stringent canonical response under the CgtA-depleted condition becomes apparent only during conditions of high growth rate. To support this statement, we showed that the mRNA expression of *cgtA* in LB is restricted to the exponential growth phase and declines in the stationary phase. Thus, CgtA is an essential gene irrespective of its role in the stringent response.

## MATERIALS AND METHODS

### Bacterial strains.

Streptomycin-resistant V. cholerae El Tor N16961 was the target for constructing the mutant strains. pKAS32 suicide vector-based constructs, which require the Pi protein for replication, were maintained in E. coli SM10λpir. Plasmids based on the pBAD18-Cm expression vector were maintained in E. coli DH5ɑ. To facilitate bipartite mating, E. coli SM10λpir was used as a donor strain, and V. cholerae El Tor N16961 was used as a recipient strain. Cells were cultured in lysogeny broth (LB) or solidified medium (1.5% agar) at 37°C. A high concentration of streptomycin antibiotic was incorporated into LB agar media to facilitate the counterselection process. Thiosulfate-citrate-bile salts-sucrose (TCBS) agar selective agar media (HiMedia) was also used to select the Vibrio cholerae cells after biparental mating. Bacterial cultures were stored in LB broth with 25% glycerol at −80°C and grown in LB broth and LB agar. Ampicillin at concentrations of 100 μg/mL and 150 μg/mL for E. coli and V. cholerae, respectively; streptomycin at a concentration of 100 μg/mL to 2 mg/mL for V. cholerae, kanamycin at a concentration of 40 μg/mL for V. cholerae, and chloramphenicol at concentrations of 34 μg/mL and 3 μg/mL for E. coli and V. cholerae, respectively, were added to the media.

### Plasmids for creating the *in vivo* mutant strains of V. cholerae.

The suicide plasmid used to create the *in vivo* mutant strains, pKAS32, contains the ampicillin marker gene, allowing antibiotic resistance selection. pKAS32 plasmid also contains the *rpsL* gene of E. coli. This locus encodes ribosomal protein S12, which is useful as a positive selection marker because mutations in the *rpsL* gene that confer streptomycin resistance (Sm^r^) are recessive in a bacterial strain that expresses Wt *rpsL* protein. The E. coli *rpsL* expressed from pKAS32 is dominant over the *rpsL* expressed from the V. cholerae genome, which aids in counterselection when plated on a high concentration of streptomycin. This will lead to the selection of colonies that have undergone an allelic exchange by homologous recombination. Also, pKAS32 contains the origin of replication, R6K, which ensures that the plasmid replication is dependent upon π, the *pir* gene product. In this study, an extra XbaI restriction site was introduced at the multiple-cloning site (MCS) of pKAS32. pKAS32-UK^r^D was created by digesting pGEMT-UK^r^D with XbaI and ligating the 2-kb insert, UK^r^D, with similarly digested pKAS32 with T4 DNA ligase. The construct for introducing the C-terminal deletion *cgtA* allele was created using a similar strategy. Competent E. coli SM10λpir was selected with ampicillin.

In order to delete *cgtA*, an essential gene, from the genome of V. cholerae, a Wt copy of *cgtA* was expressed from an arabinose-inducible araBAD promoter from a pBAD18-Cm expression vector (see Fig. S5-S7 in the supplemental material). The Wt *cgtA* copy from the genome was replaced by a kanamycin marker after homologous recombination to create a *cgtA* knockdown strain. Similarly, Wt *cgtA* from the genome was replaced by *cgtA*(ΔCTD) allele fused with a kanamycin marker. The primers for creating the constructs for full-length *cgtA* knockdown and *cgtA*(ΔCTD) are listed in Table S7A and B.

### Growth studies of *in vivo* mutant strains of V. cholerae.

In order to study the growth pattern, each V. cholerae mutant strain was subcultured in 100 mL of M9 minimal medium in 500-mL conical flasks from overnight-grown primary cultures after diluting to an optical density at 600 nm (OD_600_) of ~0.02. OD_600_ was measured at 30-min intervals during the growth. The data were plotted and analyzed statistically using GraphPad Prism 5.0 software (GraphPad Software, San Diego, CA, USA). The growth kinetics of both strains were also studied. The rate constant (*K*) and the doubling time were estimated based on the exponential growth equation

$$\text{Y}=Y_{0}\;{\text{e}}^{\mathrm{Kt}},\mathrm{where}K=\ln_{2}/t_{D}$$

where *Y*_0_ is the OD_600_ when *X* (time) is zero, *K* is the rate constant (reciprocal of the *x* axis time units in minutes), expressed in inverse minutes, tau is the time constant and the reciprocal of rate constant *K*, and *t_D_* is the doubling time, expressed as ln_2_/*K*.

### Resuscitation of persister cells.

The overnight primary cultures of all the V. cholerae strains in this study were grown without arabinose supplementation to stop the in-*trans cgtA* expression. The primary culture was washed twice in M9 minimal media, and after 1:50 times dilution, the secondary culture in M9 minimal media was inoculated. The cultures grew for 38 h (late stationary phase) at 37°C. Resuscitation studies were conducted to check if any persister cells had formed under starvation conditions. Briefly, the persister cells were resuscitated by releasing the nutritional stress at 37°C. The plates were observed at various time points of incubation. After overnight incubation, the cells were estimated by calculating the log CFU/mL. Following this, the plates were further incubated for 24 h to check for the resuscitation of the persister cell population.

### Soft agar motility assay.

The soft agar plates were incubated at 37°C for 6 h, and then we measured the motility diameter for each V. cholerae strain, including Wt, *ΔcgtA::kan^r^*, and ΔCTD *cgtA::kan^r^*. LB soft agar (1% tryptone, 0.5% NaCl, and 0.3% agar) swarm plates were used to assess the chemotactic behavior. With the help of sterile toothpicks, soft agar plates for swarming assay were inoculated by touching a bacterial colony on the LB agar plate and stabbing the toothpick into the soft agar plate. The plates were incubated at 30°C for 6 h under aerobic conditions. Plates were photographed with a Syngene G:BOX imaging station. An average of three experiments was taken to calculate the swarming diameter, followed by statistical analysis.

### Scanning electron microscopy.

All the strains of V. cholerae for this study were collected from the exponential phase either in LB broth or M9 minimal media supplemented with Casamino Acids and glucose. The cells were harvested at 6,000 rpm for 15 min. The cells were collected and washed twice with 1× phosphate-buffered saline (PBS) buffer. Then, the cells were fixed in 2.5% glutaraldehyde prepared in 1× PBS for 2 h. The sample was mixed gently by gentle pipetting for 30 min, followed by washing the cells twice with 1× PBS and serially dehydrating them with various percentages of ethanol (from 30% to 100%). The cells were then loaded onto a broken glass coverslip, dried in a vacuum, and coated with platinum. Samples were then observed under the field emission scanning electron microscope (FESEM) (model no. Supra 55 VP-4132; Carl Zeiss).

### Genome-wide stress response induced by CgtA depletion by transcriptome analysis using RNA-Seq of V. cholerae strains.

One microgram of total RNA was taken for rRNA depletion using the QIAseq FastSelect 5S/16S/23S kit (catalog no. 335925; Invitrogen) according to the manufacturer's protocol. This method offers selective depletion of bacterial 5S, 16S, and 23S rRNA. In brief, the RNA sample was hybridized to the FastSelect 5S/16S/23S probe, followed by the removal of this complex through QIAseq beads included in the kit. Thus, depleted RNA was eluted with nuclease-free water. The NEBNext Ultra II RNA library prep kit (catalog no. E7775S; New England Biolabs) for Illumina was used for the library preparation. The enriched transcriptome was chemically fragmented in a magnesium-based buffer at 94°C for 15 min. The fragmented samples were primed with random hexamers and reverse transcribed to form cDNA, and the first-strand cDNA reactions were converted to double-stranded DNA (dsDNA). The double-stranded cDNA fragments were cleaned using AMPure beads (catalog no. A63881; Beckman Coulter). The cDNA fragments undergo end repair wherein the mix converts the overhangs resulting from fragmentation into blunt ends. The end-repair mixture’s 3′ to 5′ exonuclease activity removes the 3′ overhangs, and polymerase activity fills in the 5′ overhangs. Adenylation was performed on the blunt-ended fragments by adding a single “A” nucleotide to the 3′ ends. Loop adapters (platform specific) were ligated and cleaved with the uracil-specific excision reagent (USER) enzyme to the adenylated fragments. The samples were further purified using AMPure beads. Furthermore, the DNA was amplified by 12 cycles of PCR with NEBNext Ultra II Q5 master mix and NEBNext multiplex oligonucleotides for Illumina to facilitate multiplexing while sequencing. The amplified products were then purified using 0.9× AMPure XP beads, and the final DNA library was eluted in 15 μL of 0.1× Tris-EDTA (TE) buffer. The library concentration was determined in a Qubit 3.0 fluorometer (catalog no. Q33216; Life Technologies) using the Qubit dsDNA high-sensitivity (HS) assay kit (catalog no. Q32854; Thermo Fisher Scientific). The Qubit 1× dsDNA HS assay kit contains high-sensitivity DNA reagents, buffers, and two DNA standards. DNA HS assay reagent is one of the most sensitive detection dyes for the accurate quantitation of DNA per library in solution, with linear fluorescence detection in the range of 10 pg/μL to 100 ng/μL of DNA. Dye and buffers were diluted at a 1:200 ratio, and 1 μL of the library was mixed with the dye mix and incubated at room temperature (RT) for 2 min. The readings were taken in the Qubit 3.0 fluorometer. Prior to the sample's measurement, the instrument was calibrated using the two standards provided in the kit. The library quality assessment was done using Agilent D1000 ScreenTape system (catalog no. 5067-5582; Agilent) in a 4150 TapeStation system (catalog no. G2992AA; Agilent), which is designed for analyzing DNA molecules from 100 to 1,000 bp. One microliter of the purified library was mixed with 3 μL of D1000 sample buffer (catalog no. 5067-5583), vortexed using an IKA vortexer at 2,000 rpm for 1 min, and spun down to collect the sample to the bottom of the strip. The strip was then loaded on the Agilent 4150 TapeStation system. The sequence data were generated using Illumina HiSeq. Data quality was checked using FastQC and MultiQC software. The data were checked for base call quality distribution, percentage of bases above Q20 and Q30, %GC, and sequencing adapter contamination. All the samples have passed the quantity control (QC) threshold (Q20 > 95%). Raw sequence reads were processed to remove adapter sequences and low-quality bases using fastp. The QC-passed reads were mapped onto the indexed V. cholerae reference genome (O1 biovar El Tor strain N16961) using the Bowtie 2 aligner. On average, 98.55% of the reads aligned onto the reference genome. Gene-level expression values were obtained as reading counts using featureCounts software. Differential expression analysis was carried out using the DESeq2 package. Thread counts were normalized (variance-stabilized normalized counts) using DESeq2, and differential enrichment analysis was performed. The test sample was compared to the control sample. Genes with absolute log_2_ fold change of ≥1 and *P* value of ≤0.05 were considered significant. The UpSetR R package generated plots showing significant overlapping genes between conditions. The expression profile of differentially expressed genes across the samples is presented in volcano plots and heatmaps. The genes that showed significant differential expression were used for gene ontology (GO) and pathway enrichment analysis.

### Gene ontology analysis for RNA-Seq of full-length *cgtA* knockdown strain.

The detailed gene ontology analysis for functional characterization of CgtA in V. cholerae is represented in three pie charts as molecular function, cellular components, and biological processes only for the *cgtA* knockdown versus Wt strains. The analysis was performed by a web-based server, Comparative GO (https://www.comparativego.com/) (36).

### RNA extraction for time-dependent *cgtA* expression studies by semiquantitative RT-PCR.

After shifting the cells from LB to minimal media, 1 mL of the sample was collected at 0, 1, 3, 5, 10, 15, 30, 60, 120, and 180 min. At the same time, samples were also collected from a culture growing in nutrient-rich LB media. Cells were pelleted down followed by washing with PBS by centrifugation at 6,000 rpm for 10 min. The cell pellet was resuspended in 250 μL of lysis buffer (PBS with 1% 2-mercaptoethanol) to the double suspension volume of TRIzol-chloroform (400 μL of TRIzol plus 100 μL of chloroform) and was added and mixed by vortexing and incubated in room temperature for 5 min. The mixture was centrifuged at 16,000 × *g* for 10 min at 4°C. The aqueous phase was collected, and 500 μL of chloroform was added to it. The mixture was centrifuged at 16,000 × *g* for 10 min at 4°C. The aqueous phase was collected in a fresh centrifuge tube, and 1/10 volume of 3 M sodium acetate, pH 5.2, and one volume of isopropanol were added and incubated at −20°C for 2 h. The total RNA was pelleted down by centrifugation at 16,000 × *g* for 30 min at 4°C. The pellet was then washed with 70% ethanol twice. The pellet was then air-dried and dissolved in diethyl pyrocarbonate (DEPC) water.

### RNA extraction for real-time PCR.

According to the manufacturer's protocol, the total RNA from the cells harvested from the stationary phase was isolated using Invitrogen PureLink RNA minikit (catalog no. 12183020). The concentration of total RNA was measured with a BioSpectrometer. The ratio of absorbance at 260 nm and 280 nm was found to be between 1.8 and 2.0.

### cDNA synthesis for real-time and semiquantitative RT-PCR.

For cDNA synthesis, 1 μg of RNA was treated with DNase I to eliminate any trace amount of genomic DNA contamination by incubating at 37°C for 30 min. Further, 50 mM EDTA was added to the mixture, followed by incubation at 65°C for 10 min to inactivate DNase I. The treated RNA was then used for cDNA preparation using *cgtA*-specific reverse primer (Table S8). The isolated RNA and primer were incubated at 65°C for 5 min. The mixture was quickly chilled on ice for proper primer annealing to the RNA template. The reaction was set as per the kit manual (Thermo Fisher; RevertAid first-strand cDNA synthesis kit; catalog no. K1661). It was then incubated at 25°C for 5 min, followed by 1 h of incubation at 37°C. The reaction was then terminated by heating the mixture at 70°C. For the control experiment, the reaction mixture was prepared without reverse transcriptase. 1 μL of the prepared cDNA was used to set the PCR with gene-specific primers for semiquantitative RT-PCR. The rest of the cDNA was stored at −80°C for later use. Triplicate biological samples were considered for the experiment. The PCR products were electrophoresed in 1% agarose gel and visualized by ethidium bromide solution. The images were captured by ChemiDoc G:BOX system from Syngene using the GeneSys software. The band intensity was measured by ImageJ software.

### Quantitative real-time PCR.

A master mix was prepared with 10% coverage for the appropriate number of RT-PCRs. The components were mixed thoroughly and centrifuged briefly to spin down and eliminate the air bubbles in the master mix tube. The appropriate volume of each reaction mixture was transferred to each PCR tube. The reaction tubes were transferred to the real-time PCR instrument, and the program was run according to the manufacturer's protocol (when the melting temperature [*T_m_*] of primer was ≥60°C). 16S rRNA is the housekeeping gene for performing real-time quantitative PCR. A no-template control (NTC) reaction was kept as a negative control in which all components except cDNA were added to check whether there was any contaminating source. Another control called “minus RT control” was also kept. The reverse transcription reaction was set without the reverse transcriptase enzyme to check amplification from chromosomal genes due to genomic DNA (gDNA) contamination in the RNA sample. The threshold cycle (ΔΔ*C_T_*) value was calculated for both the experimental and the control samples, followed by ΔΔ*C_T_* value calculation. The fold change in gene expression is expressed as 2^–ΔΔ^*^CT^*. The ΔΔ*C_T_* method, also known as the 2^–ΔΔ^*^CT^* method, is a formula used to calculate the relative fold gene expression of samples when performing RT-PCR. The gene-specific primers for qRT-PCR are listed in Table S8.

### Quantitative label-free proteomics.

Label-free proteomics was conducted to acquire first-hand knowledge of the cellular protein abundance scenario under the *cgtA*-depleted and CgtA(ΔCTD) conditions. The experiment was performed in three biological replicates of V. cholerae strains, including Wt, Δ*cgtA::kan^r^* V. cholerae, and ΔCTD *cgtA::kan^r^* V. cholerae. Single colonies of each of the V. cholerae strains grown on LB agar plates were inoculated in LB broth with 0.1% arabinose overnight. The cultures were washed in M9 minimal media twice and diluted (1:50) into fresh M9 minimal media supplemented with Casamino Acids. The cells were harvested in the mid-logarithmic phase and lysed to obtain the protein extracts. Twenty-five microliters of extracted protein samples were taken and reduced with 5 mM Tris(2-carboxyethyl)phosphine hydrochloride (TCEP), alkylated with 50 mM iodoacetamide, and then digested with trypsin (1:50, trypsin/lysate ratio) for 16 h at 37°C. Digests were cleaned using a C_18_ silica cartridge to remove the salt and dried using a SpeedVac. The dried pellet was resuspended in buffer A (2% acetonitrile, 0.1% formic acid). Experiments were performed on an Ultimate 3000 RSLCnano system coupled with a Thermo Q Exactive Plus. One microgram was loaded on a C_18_ 50-cm, 3.0-μm Easy-Spray column (Thermo Fisher Scientific). Peptides were eluted with a 0 to 40% gradient of buffer B (80% acetonitrile, 0.1% formic acid) at a flow rate of 300 nL/min and injected for mass spectrometry (MS) analysis. The liquid chromatography gradients were run for a total period of 100 min. The MS1 spectra were acquired in the Orbitrap at 70,000 resolution. Dynamic exclusion was employed for 10 s, excluding all charge states for a given precursor. The MS2 spectra were acquired at 17,500 resolution.

The RAW files generated after sample processing were analyzed with the software Proteome Discoverer 2.2 against the UniProt proteome database. The two algorithms used to identify the peptides from the mass spectrometry data are SEQUEST and Amanda. SEQUEST is a gold-standard algorithm for the accurate identification of peptides. In comparison, Amanda is very robust due to the high speed of spectrum identification. Both algorithms are used synchronously to get a high overlap of identified spectra of peptides. The precursor and fragment mass tolerances for both algorithms were set at 10 ppm and 0.02 Da, respectively.

All statistical analyses were performed using filtered raw abundance values based on valid values followed by log_2_ transformation. Missing values were imputed using the normal distribution of the data. The imputed data set was further standardized using Z-score. Z-data give an idea about the distance between the data point and the mean. They are used to standardize a distribution. If the value of the Z-score is above the mean, then its value is positive. If the value of the Z-score is below the mean, then its value is negative. ANOVA test was used for all three conditions, Wt, full-length CgtA depletion, and C-terminal domain truncated condition. Student’s *t* test was used to compare Wt and full-length CgtA depletion or Wt and CgtA(ΔCTD) conditions. Significance was calculated using *q* values (corrected *P* values) using a cutoff value of 0.05.

### Descriptive statistics of the differentially regulated proteomics identified by label-free proteomics.

The proteins identified by label-free proteomics were statistically validated by ANOVA. The ANOVA test is used to validate if the means of more than two groups are statistically significant from each other. The proteins upregulated and downregulated were represented through a heatmap. A heatmap is a two-dimensional representation of big data for an immediate visual summary of the information. The magnitude of the protein expression levels of the ANOVA-significant proteins is depicted as colored grids. The Z-score of protein abundance values ranged from −2 to +2. After the ANOVA statistical test, the significantly altered proteins (up- and downregulated) were sorted by the *P* value. The list of altered proteins was further sorted based on the fold change (abundance ratio). The fold change refers to the ratio of the abundance of a protein under the mutant condition to the Wt condition. A less stringent cutoff was applied that would capture more altered proteins. The cutoff value for sorting proteins based on the fold change for the significantly upregulated proteins is ≥1.2 [since log_2_(1.2) = 0.26] (37). whereas, for the significantly downregulated proteins, the cutoff value for fold change is ≤0.83 [since log_2_(0.83) = −0.26] (38). On the logarithmic scale, the significantly altered proteins with a log_2_ fold change between +0.26 and −0.26 are not considered for further analysis.

### Protein-protein interaction network.

The protein-protein interaction network presents a conceptual framework for an enhanced understanding of the functional organization of the proteome. After statistical enrichment of the altered proteins under both the *cgtA*-depleted and CgtA(ΔCTD) conditions, the protein-protein interaction networks were analyzed. For this, the STRING prediction server was used (https://string-db.org/). STRING is essential for predicting direct (physical) and indirect (functional) protein-protein associations. After STRING predicted the protein-protein interaction network, the interactive maps were visualized by open-source software, Cytoscape (https://cytoscape.org/). The layout for protein-protein interaction visualization is the attribute circle layout. All the nodes in the network are located around a circle.

### Statistical analyses.

GraphPad Prism 9 was used for statistical analysis (GraphPad Inc., San Diego, CA; http://www.graphpad.com) A *t* test has been performed to determine the significance of the data; *, *P* < 0.05; **, *P* < 0.01; and ***, *P* < 0.001.

### Data availability.

Further information and requests for resources and reagents should be directed to and will be fulfilled by the corresponding author. There is no restriction on materials generated for this study and first reported here. The accession numbers for the data reported in this paper are as follows. The data for this study have been deposited in the European Nucleotide Archive (ENA) at EMBL-EBI under BioProject accession number PRJEB53015 (SRA accession number ERP137772) (https://www.ebi.ac.uk/ena/browser/view/PRJEB53015). The mass spectrometry proteomics data have been deposited to the ProteomeXchange Consortium via the PRIDE (39) partner repository with the data set identifier PXD034015. All other data are available from the corresponding author upon request.

## References

[1] Lutz C, Erken M, Noorian P, Sun S, McDougald D. 2013. Environmental reservoirs and mechanisms of persistence of Vibrio cholerae. Front Microbiol 4:375. doi:10.3389/fmicb.2013.00375. 24379807 PMC3863721

[2] Jiang M, Sullivan SM, Wout PK, Maddock JR. 2007. G-protein control of the ribosome-associated stress response protein SpoT. J Bacteriol 189:6140–6147. doi:10.1128/JB.00315-07. 17616600 PMC1951942

[3] Sinha AK, Winther KS, Roghanian M, Gerdes K. 2019. Fatty acid starvation activates RelA by depleting lysine precursor pyruvate. Mol Microbiol 112:1339–1349. doi:10.1111/mmi.14366. 31400173

[4] Pal RR, Das B, Dasgupta S, Bhadra RK. 2011. Genetic components of stringent response in Vibrio cholerae. Indian J Med Res 133:212–217. 21415497 PMC3089054

[5] Raskin DM, Judson N, Mekalanos JJ. 2007. Regulation of the stringent response is the essential function of the conserved bacterial G protein CgtA in Vibrio cholerae. Proc Natl Acad Sci USA 104:4636–4641. doi:10.1073/pnas.0611650104. 17360576 PMC1838653

[6] Kobayashi G, Moriya S, Wada C. 2001. Deficiency of essential GTP-binding protein CgtA in Escherichia coli inhibits chromosome partition. Mol Microbiol 41:1037–1051. doi:10.1046/j.1365-2958.2001.02574.x. 11555285

[7] Sikora AE, Zielke R, Wegrzyn A, Wegrzyn G. 2006. DNA replication defect in the Escherichia coli cgtA(ts) mutant arising from reduced DnaA levels. Arch Microbiol 185:340–347. doi:10.1007/s00203-006-0099-3. 16518617

[8] Lin B, Thayer DA, Maddock JR. 2004. The Caulobacter crescentus CgtAC protein cosediments with the free 50S ribosomal subunit. J Bacteriol 186:481–489. doi:10.1128/JB.186.2.481-489.2004. 14702318 PMC305748

[9] Chatterjee A, Datta PP. 2015. Two conserved amino acids of juxtaposed domains of a ribosomal maturation protein CgtA sustain its optimal GTPase activity. Biochem Biophys Res Commun 461:636–641. doi:10.1016/j.bbrc.2015.04.079. 25912137

[10] Jiang M, Datta K, Walker A, Strahler J, Bagamasbad P, Andrews PC, Maddock JR. 2006. The Escherichia coli GTPase CgtAE is involved in late steps of large ribosome assembly. J Bacteriol 188:6757–6770. doi:10.1128/JB.00444-06. 16980477 PMC1595513

[11] Maouche R, Burgos HL, My L, Viala JP, Gourse RL, Bouveret E. 2016. Coexpression of Escherichia coli obgE, encoding the evolutionarily conserved Obg GTPase, with ribosomal proteins L21 and L27. J Bacteriol 198:1857–1867. doi:10.1128/JB.00159-16. 27137500 PMC4907111

[12] Feng B, Mandava CS, Guo Q, Wang J, Cao W, Li N, Zhang Y, Zhang Y, Wang Z, Wu J, Sanyal S, Lei J, Gao N. 2014. Structural and functional insights into the mode of action of a universally conserved Obg GTPase. PLoS Biol 12:e1001866. doi:10.1371/journal.pbio.1001866. 24844575 PMC4028186

[13] Wout P, Pu K, Sullivan SM, Reese V, Zhou S, Lin B, Maddock JR. 2004. The Escherichia coli GTPase CgtAE cofractionates with the 50S ribosomal subunit and interacts with SpoT, a ppGpp synthetase/hydrolase. J Bacteriol 186:5249–5257. doi:10.1128/JB.186.16.5249-5257.2004. 15292126 PMC490892

[14] Pletzer D, Blimkie TM, Wolfmeier H, Li Y, Baghela A, Lee AHY, Falsafi R, Hancock REW. 2020. The stringent stress response controls proteases and global regulators under optimal growth conditions in Pseudomonas aeruginosa. mSystems 5:e00495-20. doi:10.1128/mSystems.00495-20. PMC7406228 32753509

[15] Fossum S, Crooke E, Skarstad K. 2007. Organization of sister origins and replisomes during multifork DNA replication in Escherichia coli. EMBO J 26:4514–4522. doi:10.1038/sj.emboj.7601871. 17914458 PMC2063475

[16] Wang M, Chan EWC, Wan Y, Wong MH, Chen S. 2021. Active maintenance of proton motive force mediates starvation-induced bacterial antibiotic tolerance in Escherichia coli. Commun Biol 4:1068. doi:10.1038/s42003-021-02612-1. 34521984 PMC8440630

[17] Torrent M, Chalancon G, de Groot NS, Wuster A, Madan Babu M. 2018. Cells alter their tRNA abundance to selectively regulate protein synthesis during stress conditions. Sci Signal 11:eaat6409. doi:10.1126/scisignal.aat6409. 30181241 PMC6130803

[18] Dryselius R, Izutsu K, Honda T, Iida T. 2008. Differential replication dynamics for large and small Vibrio chromosomes affect gene dosage, expression and location. BMC Genomics 9:559. doi:10.1186/1471-2164-9-559. 19032792 PMC2612033

[19] Ortega DR, Kjaer A, Briegel A. 2020. The chemosensory systems of Vibrio cholerae. Mol Microbiol 114:367–376. doi:10.1111/mmi.14520. 32347610 PMC7534058

[20] Svenningsen SL, Kongstad M, Stenum TS, Munoz-Gomez AJ, Sorensen MA. 2017. Transfer RNA is highly unstable during early amino acid starvation in Escherichia coli. Nucleic Acids Res 45:793–804. doi:10.1093/nar/gkw1169. 27903898 PMC5314770

[21] Gkekas S, Singh RK, Shkumatov AV, Messens J, Fauvart M, Verstraeten N, Michiels J, Versées W. 2017. Structural and biochemical analysis of Escherichia coli ObgE, a central regulator of bacterial persistence. J Biol Chem 292:5871–5883. doi:10.1074/jbc.M116.761809. 28223358 PMC5392579

[22] Chatterjee A, Acharjee A, Das S, Datta PP. 2019. Deletion analyses reveal insights into the domain specific activities of an essential GTPase CgtA in Vibrio cholerae. Arch Biochem Biophys 665:143–151. doi:10.1016/j.abb.2019.03.007. 30894284

[23] Lee SA, Gallagher LA, Thongdee M, Staudinger BJ, Lippman S, Singh PK, Manoil C. 2015. General and condition-specific essential functions of Pseudomonas aeruginosa. Proc Natl Acad Sci USA 112:5189–5194. doi:10.1073/pnas.1422186112. 25848053 PMC4413342

[24] Salah Ud-Din AIM, Roujeinikova A. 2017. Methyl-accepting chemotaxis proteins: a core sensing element in prokaryotes and archaea. Cell Mol Life Sci 74:3293–3303. doi:10.1007/s00018-017-2514-0. 28409190 PMC11107704

[25] Rosario MM, Ordal GW. 1996. CheC and CheD interact to regulate methylation of Bacillus subtilis methyl-accepting chemotaxis proteins. Mol Microbiol 21:511–518. doi:10.1111/j.1365-2958.1996.tb02560.x. 8866475

[26] Ringgaard S, Schirner K, Davis BM, Waldor MK. 2011. A family of ParA-like ATPases promotes cell pole maturation by facilitating polar localization of chemotaxis proteins. Genes Dev 25:1544–1555. doi:10.1101/gad.2061811. 21764856 PMC3143943

[27] Li M, Hazelbauer GL. 2011. Core unit of chemotaxis signaling complexes. Proc Natl Acad Sci USA 108:9390–9395. doi:10.1073/pnas.1104824108. 21606342 PMC3111312

[28] Briegel A, Li X, Bilwes AM, Hughes KT, Jensen GJ, Crane BR. 2012. Bacterial chemoreceptor arrays are hexagonally packed trimers of receptor dimers networked by rings of kinase and coupling proteins. Proc Natl Acad Sci USA 109:3766–3771. doi:10.1073/pnas.1115719109. 22355139 PMC3309718

[29] Piñas GE, Frank V, Vaknin A, Parkinson JS. 2016. The source of high signal cooperativity in bacterial chemosensory arrays. Proc Natl Acad Sci USA 113:3335–3340. doi:10.1073/pnas.1600216113. 26951681 PMC4812747

[30] Yamaichi Y, Bruckner R, Ringgaard S, Möll A, Cameron DE, Briegel A, Jensen GJ, Davis BM, Waldor MK. 2012. A multidomain hub anchors the chromosome segregation and chemotactic machinery to the bacterial pole. Genes Dev 26:2348–2360. doi:10.1101/gad.199869.112. 23070816 PMC3475806

[31] Ringgaard S, Yang W, Alvarado A, Schirner K, Briegel A. 2018. Chemotaxis arrays in Vibrio species and their intracellular positioning by the ParC/ParP system. J Bacteriol 200:e00793-17. doi:10.1128/JB.00793-17. 29531180 PMC6040185

[32] Ghosal A, Babu VMP, Walker GC. 2018. Elevated levels of Era GTPase improve growth, 16S rRNA processing, and 70S ribosome assembly of Escherichia coli lacking highly conserved multifunctional YbeY endoribonuclease. J Bacteriol 200:e00278-18. doi:10.1128/JB.00278-18. 29914987 PMC6088164

[33] Uicker WC, Schaefer L, Britton RA. 2006. The essential GTPase RbgA (YlqF) is required for 50S ribosome assembly in Bacillus subtilis. Mol Microbiol 59:528–540. doi:10.1111/j.1365-2958.2005.04948.x. 16390447

[34] Irving SE, Choudhury NR, Corrigan RM. 2021. The stringent response and physiological roles of (pp)pGpp in bacteria. Nat Rev Microbiol 19:256–271. doi:10.1038/s41579-020-00470-y. 33149273

[35] Shah S, Das B, Bhadra RK. 2008. Functional analysis of the essential GTP-binding-protein-coding gene cgtA of Vibrio cholerae. J Bacteriol 190:4764–4771. doi:10.1128/JB.02021-07. 18456812 PMC2446790

[36] Fruzangohar M, Ebrahimie E, Ogunniyi AD, Mahdi LK, Paton JC, Adelson DL. 2013. Comparative GO: a web application for comparative gene ontology and gene ontology-based gene selection in bacteria. PLoS One 8:e58759. doi:10.1371/journal.pone.0058759. 23536820 PMC3594149

[37] Zhang N, Zhang L, Zhao L, Ren Y, Cui D, Chen J, Wang Y, Yu P, Chen F. 2017. iTRAQ and virus-induced gene silencing revealed three proteins involved in cold response in bread wheat. Sci Rep 7:7524. doi:10.1038/s41598-017-08069-9. 28790462 PMC5548720

[38] Zhang N, Zhang L, Shi C, Zhao L, Cui D, Chen F. 2018. Identification of proteins using iTRAQ and virus-induced gene silencing reveals three bread wheat proteins involved in the response to combined osmotic-cold stress. J Proteome Res 17:2256–2281. doi:10.1021/acs.jproteome.7b00745. 29761697

[39] Perez-Riverol Y, Bai J, Bandla C, Hewapathirana S, García-Seisdedos D, Kamatchinathan S, Kundu D, Prakash A, Frericks-Zipper A, Eisenacher M, Walzer M, Wang S, Brazma A, Vizcaíno JA. 2022. The PRIDE database resources in 2022: a hub for mass spectrometry-based proteomics evidences. Nucleic Acids Res 50:D543–D552. doi:10.1093/nar/gkab1038. 34723319 PMC8728295

