# Supplementary material for: Knockdown Experiment Reveals an Essential GTPase CgtA’s Involvement in Growth, Viability, Motility, Morphology, and Persister Phenotypes in Vibrio cholerae
Source: Microbiol Spectr. 2023 Mar 14;11(2):e03181-22. doi: 10.1128/spectrum.03181-22 (PMC10100748; doi:10.1128/spectrum.03181-22)
Supplement: Supplemental file 1 — Supplemental material. Download spectrum.03181-22-s0001.pdf, PDF file, 1.2 MB [file spectrum.03181-22-s0001.pdf]

Supplemental Material

Supplementary Tables (Table: S1-S9)

Title: Knockdown experiment reveals an essential GTPase CgtA's involvement on growth, viability, motility, morphology, and persister phenotypes in *Vibrio cholerae*

Sagarika Das[1], Ananya Chatterjee[1,2], Partha Pratim Datta[1*]

[1]Department of Biological Sciences, Indian Institute of Science Education and Research Kolkata, Mohanpur, Nadia, West Bengal, India, PIN: 741246

[2]Viral Research and Diagnostic Laboratories, National Institute of Cholera and Enteric Diseases, P-33, C.I.T. Road, Scheme XM, Beliaghata, Kolkata, West Bengal, India, PIN: 700010 (Present Address)

*Corresponding author: partha_datta@iiserkol.ac.in

Table S1A. List of altered proteins involved in chemotaxis and flagellar motility in full-length CgtA depletion condition

Accession Number	Description	Abundance Ratio	*p*-value
**Chemotaxis**			
Q9KMX7	CheC, inhibitor of MCP methylation	1.222	0.04532469
Q9KQD7	Chemotaxis protein CheA	0.274	0.00225451
Q9KKK8	Chemotaxis protein CheY	0.242	0.00620673
Q9KRN4	Chemotaxis protein CheV	0.622	0.04564578
Q9KUY1	CheX domain-containing protein	0.128	0.03179601
Q9KLT3	Methyl-accepting chemotaxis protein	3.874	0.00318864
Q9KMZ0	Methyl-accepting chemotaxis protein	0.619	0.03420517
Q9KQ43	Methyl-accepting chemotaxis protein	0.228	0.00475943
Q9KS47	Methyl-accepting chemotaxis protein	0.177	0.00879794
Q9KS54	Methyl-accepting chemotaxis protein	0.398	0.00277208
Q9KS57	Methyl-accepting chemotaxis protein	0.298	0.0011602
Q9KSF8	Methyl-accepting chemotaxis protein	0.283	0.00944832
Q9KSK8	Methyl-accepting chemotaxis protein	0.446	0.00042032
Q9KUK6	Methyl-accepting chemotaxis protein	3.065	0.04228211
P15492	Methyl-accepting chemotaxis protein HlyB	0.332	0.01522417
**Components of Flagella**			
P0C6C6	Flagellin D	0.742	0.04335798
P0C6C5	Flagellin C	0.622	0.03186311
P0C6C4	Flagellin B	0.396	0.00041126
Q9KQ76	Flagellar motor switch protein FliN	0.577	0.01843227
Q9X4Q9	Flagellar motor switch protein FliG	0.199	0.00100791

Table S1B List of altered proteins involved in cell division and DNA Replication

Accession Number	Description	Abundance Ratio	*p*-values
**Cell Division**			
Q9KQN7	Cell division topological specificity factor	0.448	0.0077348
Q9KP95	Cell division protein ZapA	0.276	0.0014913
Q9KNQ5	Cell division protein FtsN, putative	0.468	0.0339693
Q9KS02	Macrodomain Ter protein	0.66	0.0252549
**DNA Replication**			
Q9KVX5	Beta sliding clamp	7.103	0.0240692
Q9KVX6	Chromosomal replication initiator protein DnaA	2.84	0.0349008
A0A5P1R5X2	DNA topoisomerase (ATP-hydrolysing) (Fragment)	4.872	0.0131098
Q9KKJ3	ParB family protein	0.5	0.003256
Q9KNG6	ParA family protein	0.525	0.0120041
**Q9KKM8**	PEGA domain-containing protein	0.753	0.0140468
Q9KLM6	DNA helicase	2.989	0.0313502

Table S1C. List of altered proteins involved in DNA recombination and repair

Accession Number	Description	Abundance Ratio	*p*-values
**DNA Recombination**			
Q9KT49	Recombination protein RecR	0.44	0.004659
Q9KQT4	Integration host factor subunit beta	0.205	0.0004602
Q9KSN4	Integration host factor subunit alpha	1.27	0.0320394
**DNA repair**			
Q9KNV6	Phosphoglycolate phosphatase	0.713	0.0069456
Q9KRQ1	Catalase	5.17	0.0009209
Q9KUI6	DNA mismatch repair protein MutS	3.437	0.0320238
Q9KQY7	Exodeoxyribonuclease III	1.817	0.0470798
Q9KLZ7	MutT/nudix family protein	13.727	1.604E-05
Q9KSB6	MutT/nudix family protein	5.854	0.0009852

Table S2. Common chemotaxis and flagellar motility proteins significantly downregulated in both the conditions: full-length *cgtA* knockdown and CgtA(ΔCTD) deletion

Protein ID	Protein Name	Abundance Ratio (Knockdown) / (WT)	Abundance Ratio (CTD) / (WT)
Q9KS47	Methyl-accepting chemotaxis protein II (mcp-II) (aspartate chemoreceptor protein)	0.177	0.238
Q9KS54	Methyl-accepting chemotaxis sensor/transducer protein	0.398	0.39
Q9KS57	Methyl-accepting chemotaxis transducer dimer protein	0.298	0.503
Q9KSF8	Methyl-accepting chemotaxis sensor/transducer protein	0.283	0.59
Q9KSK8	Methyl-accepting chemotaxis sensor/transducer protein	0.446	0.607
Q9KQ43	Methyl-accepting chemotaxis sensor/transducer protein	0.228	0.682
P15492	Methyl-accepting chemotaxis protein, hemolysin secretion protein HylB	0.332	0.52
Q9KKK8	Chemotaxis regulator - transmits chemoreceptor signals to flagellar motor components CheY	0.242	0.581
Q9KUY1	Chemotaxis protein CheX	0.128	0.526
Q9X4Q9	Flagellar motor switch protein FliG	0.199	0.408
Q9KQ76	Flagellar motor switch protein FliN	0.577	0.677
P0C6C4	Flagellin B	0.396	0.775

Table S3. Biological functions commonly downregulated in full-length CgtA deletion and ΔCTD CgtA conditions

Protein ID	Protein Name	Abundance Ratio (Knockdown/ WT)	Abundance Ratio (ΔCTD CgtA) / (WT)
Q9KNR2	5-carboxymethyl-2-hydroxymuconate delta isomerase	0.174	0.772
Q9KKR8	Extracellular solute-binding protein, putative	0.082	0.349
Q9KUY1	CheX domain-containing protein	0.128	0.526
Q9KMW9	GMP reductase	0.183	0.712
Q9KL41	Heme oxygenase HutZ	0.196	0.75
H9L4Q3	Hcp protein	0.196	0.75
Q9KM13	Heme transport protein HutA	0.197	0.744
Q9KL36	Hemin ABC transporter, periplasmic hemin-binding protein HutB	0.101	0.357
Q9KSQ9	Uncharacterized protein	0.119	0.36
Q9KQ43	Methyl-accepting chemotaxis protein	0.228	0.682
Q9KSD3	Autoinducer 2-binding periplasmic protein LuxP	0.223	0.666
Q9KND3	Uncharacterized protein	0.2	0.589
Q9KS97	Amino acid ABC transporter, periplasmic amino acid-binding protein	0.286	0.835
Q9KKP8	NAD(P)-bd_dom domain-containing protein	0.264	0.736
Q9KP65	Aspartate carbamoyltransferase regulatory chain	0.27	0.707
Q9KPF0	Thiol:disulfide interchange protein	0.162	0.405
Q9KKK8	Chemotaxis protein CheY	0.242	0.581
Q9KQD7	Phosphorelay protein LuxU	0.274	0.64
Q9KU34	Phospho-2-dehydro-3-deoxyheptonate aldolase	0.2	0.452
Q9KPP0	Uncharacterized protein	0.112	0.243
Q9KSF8	Methyl-accepting chemotaxis protein	0.283	0.59
Q9KNP9	Met repressor	0.387	0.785
Q9KT04	Uncharacterized protein	0.21	0.421
P0C6C4	Flagellin B	0.396	0.775
Q9KN87	Uncharacterized protein	0.328	0.603
E5EUF9	Malate dehydrogenase (Fragment)	0.432	0.775
Q9KS57	Methyl-accepting chemotaxis protein	0.298	0.503
P15492	Methyl-accepting chemotaxis protein HlyB	0.332	0.52
Q9KNR7	60 kDa chaperonin 1	0.57	0.799
Q9KNW5	DUF1338 domain-containing protein	0.339	0.473
Q9F854	Histidinol dehydrogenase	0.235	0.325
Q9KSX6	DNA-binding protein	0.563	0.778
Q9KSK8	Methyl-accepting chemotaxis protein	0.446	0.607
Q9KS47	Methyl-accepting chemotaxis protein	0.177	0.238
Q9KNM3	DNA-directed RNA polymerase subunit omega	0.513	0.688
Q9KVR3	Universal stress protein	0.414	0.554
Q9KPU3	3,4-dihydroxy-2-butanone 4-phosphate synthase	0.609	0.809
Q9KTD7	Phosphoenolpyruvate-protein phosphotransferase	0.595	0.79
Q9KQ39	Arsenate reductase	0.479	0.616
P0C6C5	Flagellin C	0.622	0.783
Q9KUF5	Periplasmic serine endoprotease DegP-like	0.617	0.773
P0C6Q6	Outer membrane protein U	0.159	0.199
Q9KV51	Thioredoxin	0.622	0.765
Q9KSP7	Elongation factor P-like protein	0.666	0.798
Q9KVG0	Glutathione reductase	0.502	0.6
Q9KNX6	FKBP-type peptidyl-prolyl cis-trans isomerase SlyD	0.453	0.505
Q9K2J6	Uncharacterized protein	0.638	0.701
Q9KV04	Peptidyl-prolyl cis-trans isomerase	0.228	0.249

Q9KSA9	Glutathione-dependent peroxiredoxin	0.629	0.662
Q9KQ30	5'-nucleotidase	0.496	0.508
Q9KP95	Cell division protein ZapA	0.276	0.282
Q9KLQ3	Phasin_2 domain-containing protein	0.511	0.505
Q9KS54	Methyl-accepting chemotaxis protein	0.398	0.39
Q9KL13	Peptidase_M14 domain-containing protein	0.672	0.645
Q9KQU3	Alanine dehydrogenase	0.77	0.729
Q9KST6	Tryptophan synthase beta chain	0.747	0.673
Q9KQ76	Flagellar motor switch protein FliN	0.747	0.673
Q9KV79	Transcriptional regulator, MerR family	0.369	0.328
Q9KNU5	Penicillin-binding protein 1A	0.482	0.408
Q9X4Q9	Flagellar motor switch protein FliG	0.482	0.408
Q9KVH8	Cytochrome c5	0.326	0.271
Q9KUQ4	Pyridoxal phosphate homeostasis protein	0.589	0.469
Q9KUQ9	dITP/XTP pyrophosphatase	0.459	0.365
Q9KQX6	UPF0304 protein	0.512	0.397
Q9KRG4	Phage shock protein A	0.457	0.337
Q9KSP8	UPF0263 protein	0.51	0.372
Q9KUY4	Glucose-6-phosphate isomerase	0.811	0.57
Q9KT56	Transcriptional regulator, LysR family	0.669	0.445
Q9KUH1	Oxaloacetate decarboxylase, alpha subunit	0.671	0.388
Q9KVR1	Ferritin	0.267	0.128
Q56652	Maltoporin	0.681	0.326
Q9KMK6	Uncharacterized protein	0.83	0.354
Q9KPE3	Dephospho-CoA kinase	0.42	0.155

Table S4. Proteins significantly downregulated in ΔCTD CgtA condition but significantly upregulated or unaltered in CgtA depleted condition

Protein ID	Protein name	Abundance Ratio: (Knockdown/WT)	Abundance Ratio:(ΔCTD CgtA/WT)
Q9KU58	Uncharacterized protein	17.722	0.367
Q9KTK0	NADPH-dependent 7-cyano-7-deazaguanine reductase	3.784	0.799
Q9KPK4	Homoserine kinase	3.214	0.681
Q9KTH2	Hemolysin-related protein	3.118	0.78
Q9KLA3	Glyceraldehyde 3-phosphate dehydrogenase	2.488	0.685
Q9KVI9	Vitamin B12 transporter BtuB	1.644	0.51
Q9KS46	Metal-dependent carboxypeptidase	2.412	0.751
Q9KUJ8	Beta-ketoadipate enol-lactone hydrolase	2.084	0.711
Q9KTJ5	Methionine import ATP-binding protein MetN	1.674	0.643
Q9KPB3	GTPase Era	1.562	0.652
Q9KKR7	Methyl-accepting chemotaxis protein	0.84	0.367
Q9KL54	Probable phosphatase	1.69	0.771
Q9KQ06	Chemotaxis protein methyltransferase 1	1.03	0.553
Q9KL97	Uncharacterized protein	1.134	0.617
Q9KMX7	Response regulator	1.222	0.698
Q9KQH6	Malonyl CoA-acyl carrier protein transacylase	1.133	0.763

Table S5. Significantly altered proteins of the protein translation apparatus during CgtA depleted

Accession Number	Description	Abundance Ratio	*p*-values
**Ribosomal Proteins**			
Q9KUY9	50S ribosomal protein L9	1.798	0.0236729
Q9KNZ6	50S ribosomal protein L5	1.606	0.0058152
P0A497	50S ribosomal protein L36	4.584	0.0176792
Q9KVY1	50S ribosomal protein L34	2.68	0.002264
Q9KQH3	50S ribosomal protein L32	0.423	0.0316759
Q9KNY4	50S ribosomal protein L3	2.109	0.0165819
Q9KUS9	50S ribosomal protein L27	1.244	0.0313112
Q9KNZ5	50S ribosomal protein L24	1.377	0.0016435
P0A479	50S ribosomal protein L20	2.513	0.002759
Q9KP00	50S ribosomal protein L18	1.737	0.0087329
Q9KP09	50S ribosomal protein L17	2.453	0.0036984
Q9KNZ4	50S ribosomal protein L14	1.973	0.0095421
Q9KV34	50S ribosomal protein L11	2.14	0.0044484
Q9KUF0	30S ribosomal protein S9	1.515	0.0271653
Q9KUZ2	30S ribosomal protein S6	1.333	0.0107026
Q9KUZ0	30S ribosomal protein S18	2.149	0.0313782
Q9KU77	30S ribosomal protein S15	2.013	0.0033505
Q9KNZ7	30S ribosomal protein S14	1.463	0.0185837
**Ribosome Maturation Factors**			
Q9KPB3	GTPase Era	1.562	0.0030628
Q9KTW7	GTPase Der	3.283	0.0101291
Q9KM60	Ribosome biogenesis GTPase A	1.604	0.0459668
Q9KUF9	Ribosome maturation factor RimM	3.363	0.0068012
**Initiation, Elongation, Release, Recycling factors**			
Q9KM19	Sui1 family protein	2.135	0.0134214
Q9KPV5	Ribosome-recycling factor	0.638	0.0102554
Q9KU31	Energy-dependent translational throttle protein EttA	1.723	0.0351972
Q9KSP7	Elongation factor P-like protein	0.666	0.0026319
Q9KU64	Peptide chain release factor 3	1.95	0.0375525
**tRNA Modification**			
Q9KPA1	tRNA-modifying protein YgfZ	1.759	0.0478551
Q9KUX9	tRNA-dihydrouridine(20/20a) synthase	5.29	0.00513
Q9KS29	tRNA-cytidine (32) 2-sulfurtransferase	3.093	0.0078521
Q9KVY5	tRNA modification GTPase MnmE	3.454	0.0088212
Q9KUF8	tRNA (guanine-N (1)-)-methyltransferase	3.081	0.0242853
Q9KKP3	Putative pseudouridine methyltransferase	6.475	0.02158
**rRNA Modification**			
Q9KVJ7	Ribosomal RNA small subunit methyltransferase D	3.035	0.028277
Q9KTX3	Dual-specificity RNA methyltransferase RlmN	13.823	0.0018674
Q9KTF1	Ribosomal RNA large subunit methyltransferase H	4.48	0.0100019
Q9KNW0	Ribosomal protein S12 methylthiotransferase RimO	4.202	0.0019463
Q9KSS7	Ribosomal large subunit pseudouridine synthase B	3.842	0.0209209
Q9KPY9	Pseudouridine synthase	6.069	0.0001437
Q9KU66	[Ribosomal protein S18]-alanine N-acetyltransferase	5.279	0.0335686

**Aminoacyl tRNA synthetase**			
Q9KTA6	Glutamine--tRNA ligase	2.056	0.03445
Q9KTX0	Histidine--tRNA ligase	1.626	0.0218382

Table S6. Details of the significant differential regulation at both mRNA and protein expression levels of the 30 genes

Gene and protein identifier		Protein expression		mRNA expression	
**Gene ID**	**Protein ID**	**Log$_2$ Fold Change**	***p*-value**	**Log$_2$ Fold Change**	***p*-value**
VC0076	Q9KVR3	-1.8843944	3.02907E-06	-1.38210521	0.032306104
VC0078	Q9KVR1	-1.26908475	0.041532642	-1.60504174	0.044440197
VC0273	Q9KV83	-1.76996957	0.009635752	-1.24089249	0.049213708
VC0695	Q9KU34	-2.17897034	0.001315979	-2.10970879	0.026408432
VC0737	Q9KTZ3	-1.84103166	0.004740459	-1.62417002	0.025629973
VC0806	Q9KTT2	1.796879982	0.018408293	1.439629281	0.048486597
VC0854	O30862	-1.30135939	0.028403638	1.815102428	0.040529914
VC0930	Q9KTH2	2.112625241	0.00123491	1.770277461	0.039441036
VC1249	Q9KSK7	-1.70137863	0.038996063	-1.36657789	0.032129079
VC1298	Q9KSF8	-1.95295584	6.69473E-05	-2.83683777	0.018372752
VC1325	Q9KSD3	-2.05604474	0.006296326	-2.02047862	0.017982834
VC1362	Q9KS97	-2.14430162	0.000144864	-1.94337726	0.039401561
VC1403	Q9KS57	-2.08110174	0.002389324	-1.93905067	0.019140846
VC1607	Q9KRM9	1.352318913	0.002923016	-1.21770881	0.044233839
VC1914	Q9KQT4	-1.63574571	0.00451718	-1.31969749	0.045643171
VC2296	Q9KPS0	1.707557817	0.011657477	-1.26894	0.039512763
VC2326	Q9KPP0	-1.78433722	0.008289489	-1.40274321	0.0406664
VC2435	Q9KPD5	1.543441753	0.011481759	-1.46729951	0.033077261
VC2559	Q9KP20	-2.07941921	0.000286175	3.513573877	0.037262181
VC2562	Q9KP17	1.82022956	0.015242464	-1.79564031	0.034777313
VC2656	Q9KNS5	-1.39310947	0.024952084	-2.34166769	0.041278251
VC2698	Q9KNN3	-1.98743011	0.001182395	-2.52800028	0.031808047
VCA0010	Q9KNF4	1.877225002	0.041021192	-1.89614697	0.018581309
VCA0032	Q9KND3	-2.1631343	0.002054179	-2.03968639	0.015611256
VCA0689	Q9KLQ3	-1.943823	0.00041941	-1.78636172	0.020959642
VCA0744	Q9KLJ9	-1.82634588	0.023257516	-3.05501609	0.011523921
VCA0900	Q9KL48	-1.75170122	0.000411203	-1.7924611	0.027407072
VCA1033	Q9KKR8	-2.04954054	0.000943005	-1.59770367	0.029356912
VCA1054	Q9KKP8	-2.21432054	0.000429244	-1.31799888	0.033810147
VCA1096	Q9KKK8	-2.00295834	0.001455822	-1.62424407	0.026450025

Table S7A: Primers for *cgtA* deletion and CgtA

Primer	Sequence (5'-3')
Upstream region FP	CGGCTC**AGATCT**A**TCTAGA** ATC AAC GCT TAA
Upstream region RP for CgtA(ΔCTD)	CAT CTA CGA ATT TCA TTT TTC CTC CAC
Upstream region RP for Δ *cgtA*	GTC AGA CCC CGT TTT TCC TCC ACT
Downstream region FP	CGA TGA GTT TTT CTA ATT AAC TTA CTG G
Downstream region RP	GCT C**TC TAG A** A GAT CAT CCC AAC TGA CGA
Kanamycin marker FP for CgtA(ΔCTD)	GCATTC**CATATG** ACGGGGTCTGACGCTCA
Kanamycin marker FP for Δ *cgtA*	GAG TGG AGG AAA AAC GGG GTC TGA C
Kanamycin marker RP for Δ *cgtA*	CCA GTA AGT TAA TTA GAA AAA CTC ATC G
cgtA FP for *CgtA(ΔCTD)*	GTG GAG GAA AAA TGA AAT TCG TAG ATG
cgtA RP for *CgtA(ΔCTD)*	GCTCTGC**CATATG**TCAATCCATGAACTCGGC

Bold character indicates the following restriction sites: AGATCT : BglII , **TCTAGA :** XbaI, **CATATG** : NdeI

Table S7B: Sequences of primers used to amplify *cgtA* to clone into pBAD18Cm vector

Primer name	Sequence
Forward primer for *cgtA*	CTC TCT **GCT AGC** AGG AGG GGA TTC CAT ATG AGT **GGA GG**A AAA **ATG** AAA T
Reverse primer for *cgtA*	GCT C**GTCGAC**TCAGTCACGAACATAGAT

Bold character indicates the following restriction sites: GCT AGC: NheI, **GGA GG**: Ribosome binding site, **ATG**: Start codon, **GTCGAC:** SalI

Table S8. Primers for qRT PCR

Primer	Sequence (5'-3')	Use
cgtA-QF	AAAACCTTGGGTACCAAGGGTGAGC	Target gene-specific
cgtA-QR	GCCAGCTTTTCACTGTACTGTTCAA	Target gene-specific
glpD-QF	ACTAAACCTGAAATGCAAATTGGTT	Target gene-specific
glpD-QR	TGTGCCAATCATCGAGTAGTCATCA	Target gene-specific
bolA-QF	AAAGTGATATGCACAACGTGCCGCG	Target gene-specific
bolA-QR	TTTCTGGACTTTCCTGATTCATTTG	Target gene-specific
cheW-QF	CAAGAAATTGTGCGTATACCCGAGA	Target gene-specific
cheW-QR	TCCTTCCTGTGGGTAAGCTTCGGAG	Target gene-specific
vieA-QF	AGATTGATCGCAGCTTTGTGCATGA	Target gene-specific
vieA-QR	CCAGCAGGTTGATCGGTAAATCGCG	Target gene-specific
rmf-QF	TCGCCTAGAAAGAGCTCAATCGCAA	Target gene-specific
rmf -QR	AGTTTATCGTTTCTAGCATCACGCC	Target gene-specific
16S rRNA-QF	TCAGCTCGTGTTGTGAAATG	Internal control
16S rRNA-QR	GTAAGGGCCATGATGACTTG	Internal control

Table S9. Statistical Analysis of the Semi quantitative PCR data from Figure 11

Null hypothesis: $\mu1=\mu2$, Alternative hypothesis: $\mu1\neq\mu2$, Level of significance (α)= 0.05, Confidence level=95%										
Sl No.	Comparison Between		Mean		Standard Deviation		P-value	N		Significance
	**S1**	**S2**	**μ1**	**μ 2**	**σ 1**	**σ 2**		**n1**	**n2**	
1	LB-0 min	M9-0 min	0.336	0.786	0.035	0.068	0.007	3	3	Yes
2	LB-1 min	M9-1 min	0.311	0.329	0.078	0.024	0.680	3	3	No
3	LB-3 min	M9-3 min	0.908	0.805	0.016	0.197	0.430	3	3	No
4	LB- 5 min	M9- 5 min	0.916	0.805	0.016	0.130	0.253	3	3	No
5	LB- 10 min	M9- 10 min	0.912	0.819	0.085	0.167	0.511	3	3	No
6	LB- 15 min	M9- 15 min	1	0.94	0	0.062	0.252	3	3	No
7	LB- 30 min	M9- 30 min	0.406	0.870	0.097	0.152	0.036	3	3	Yes
8	LB- 60 min	M9- 60 min	0.226	0.927	0.032	0.063	0.004	3	3	Yes
9	LB- 120 min	M9- 120 min	0.210	0.528	0.099	0.041	0.012	3	3	Yes
10	LB- 180 min	M9- 180 min	0.189	0.114	0.109	0.113	0.535	3	3	No

Supplemental Material

Supplementary Figures (Fig: S1 – S8)

Title: Knockdown experiment reveals an essential GTPase CgtA's involvement on growth, viability, motility, morphology, and persister phenotypes in *Vibrio cholerae*

Sagarika Das[1], Ananya Chatterjee[1,2], Partha Pratim Datta[1*]

[1]Department of Biological Sciences, Indian Institute of Science Education and Research Kolkata, Mohanpur, Nadia, West Bengal, India, PIN: 741246

[2]Viral Research and Diagnostic Laboratories, National Institute of Cholera and Enteric Diseases, P-33, CIT Road, Scheme XM, Beliaghata, P.O. Box-177, Kolkata, West Bengal, India (Present address)

*Corresponding author: partha_datta@iiserkol.ac.in

Fig. S1

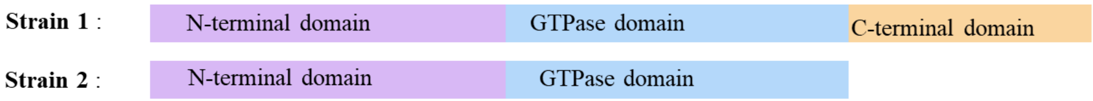

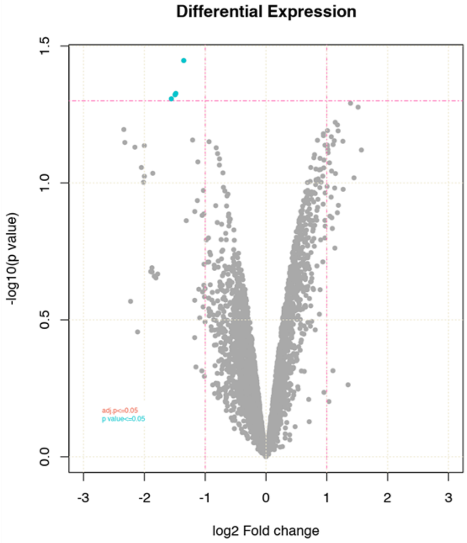

No. of genes downregulated = 4
No. of genes upregulated = 0

Volcano plot of differentially expressed genes in CgtA(ΔCTD) condition

Fig. S2

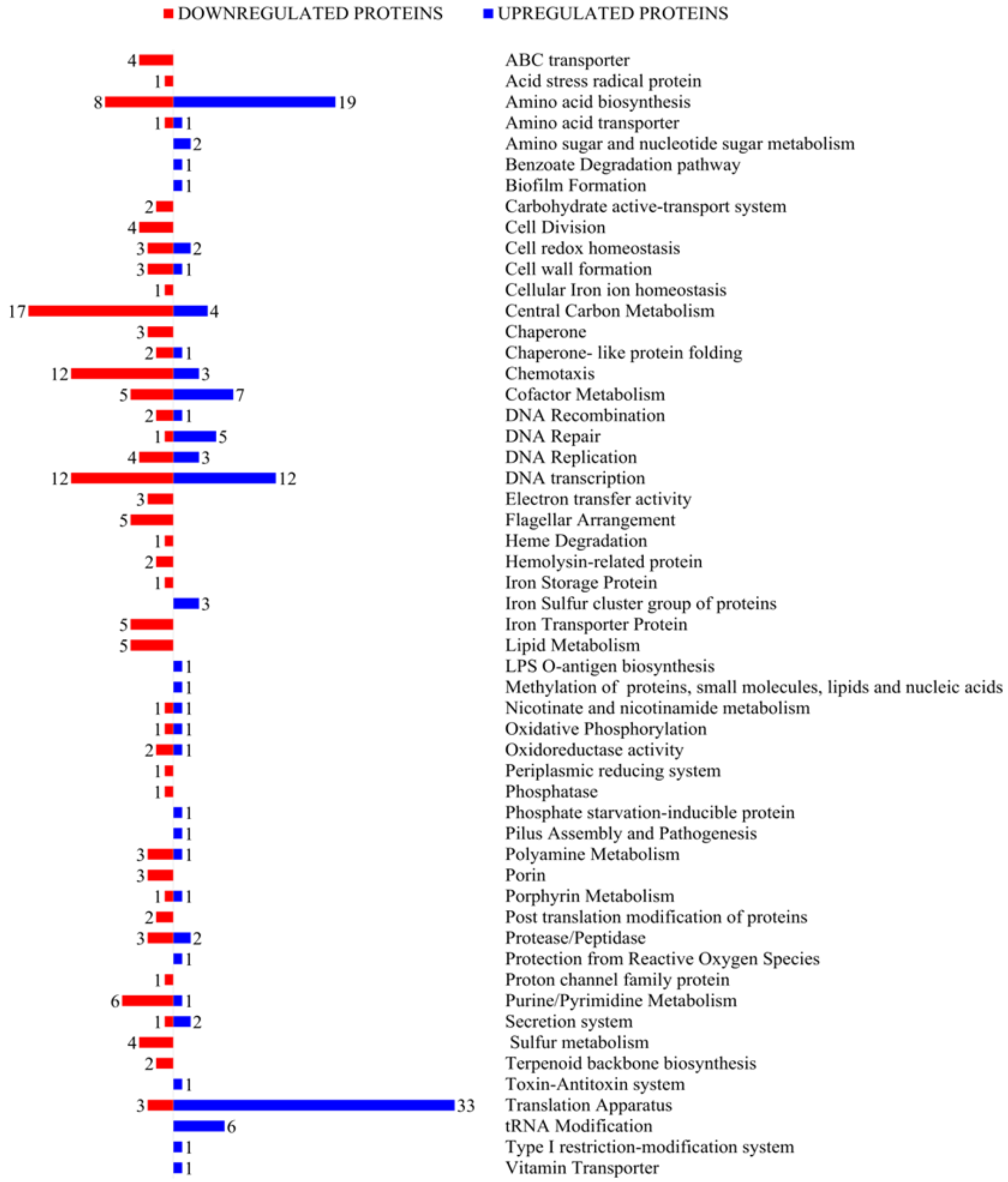

The critical biological pathways severely affected upon full-length CgtA depletion

Fig. S3

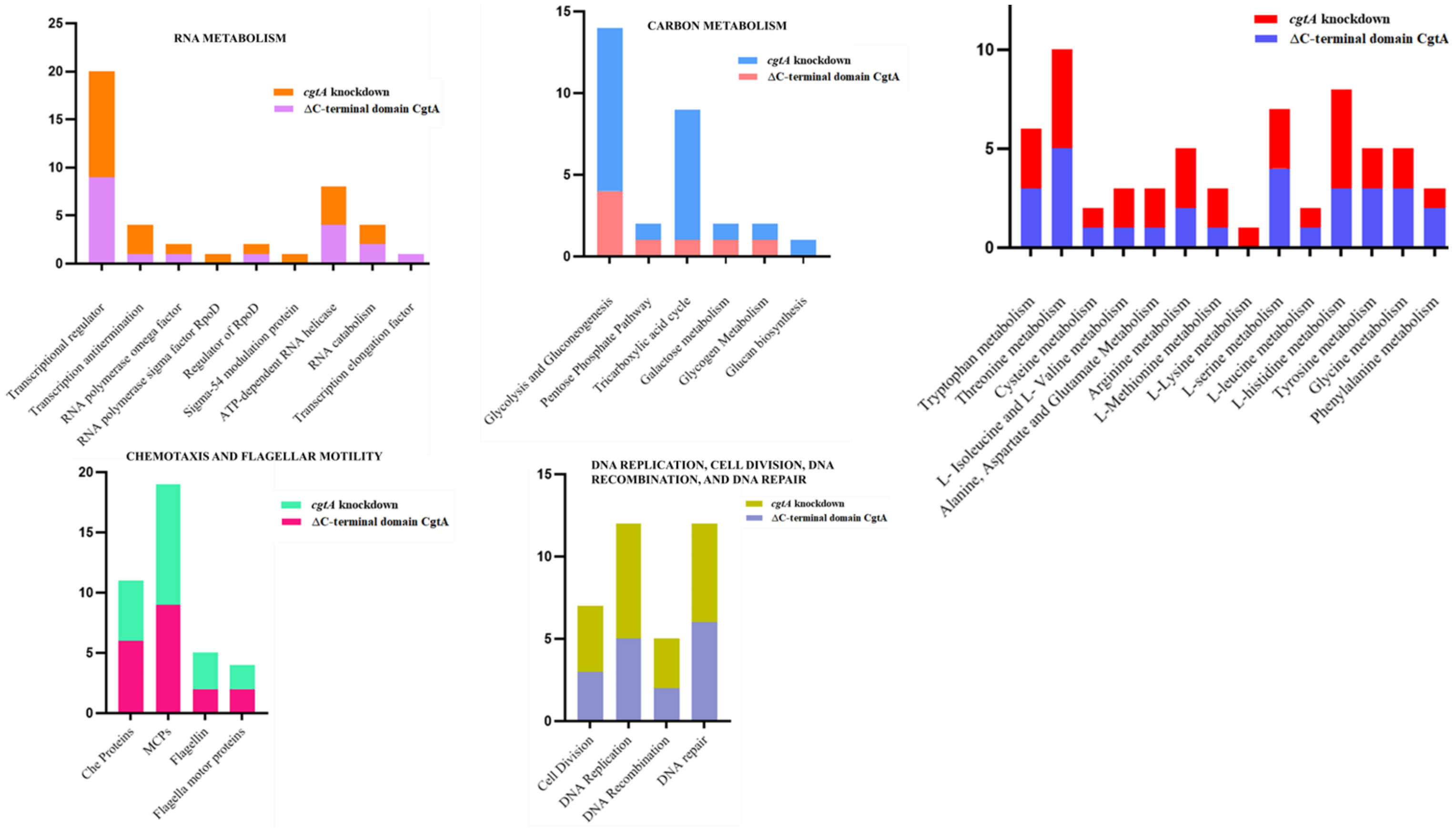

Stacked bar chart showing the distribution of proteins involved in various critical cellular processes affected in full-length CgtA depletion and CgtA(ΔCTD) conditions

Fig. S4

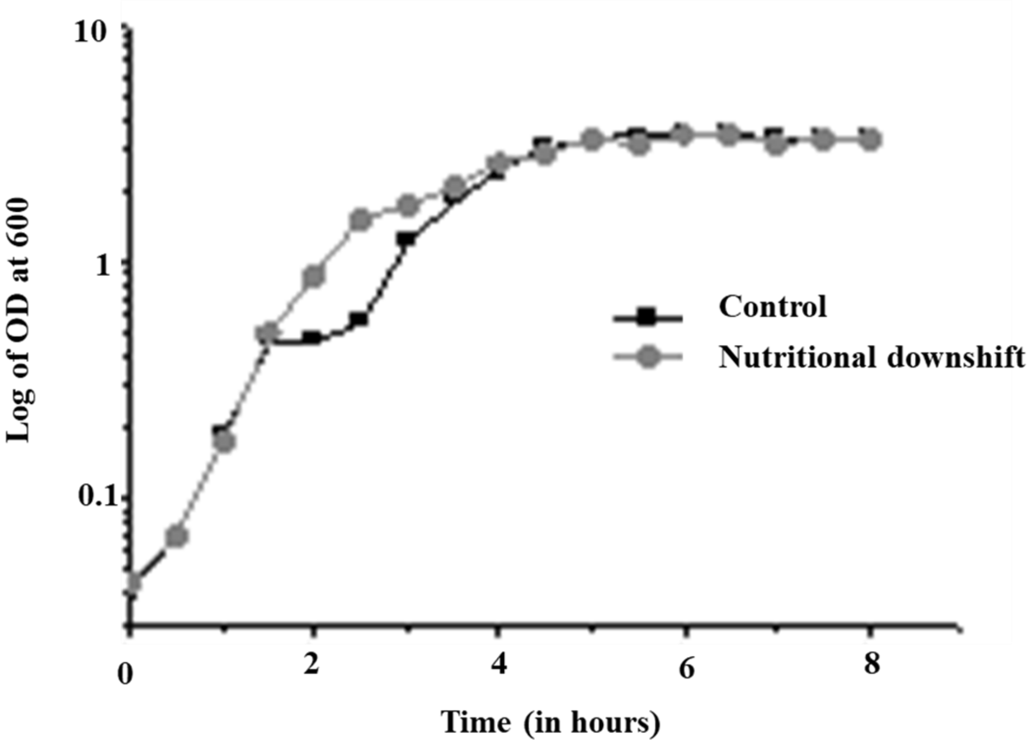

Growth curve of Wt *Vibrio cholerae* in LB media and followed by nutritional downshift in M9 minimal media

Fig. S5

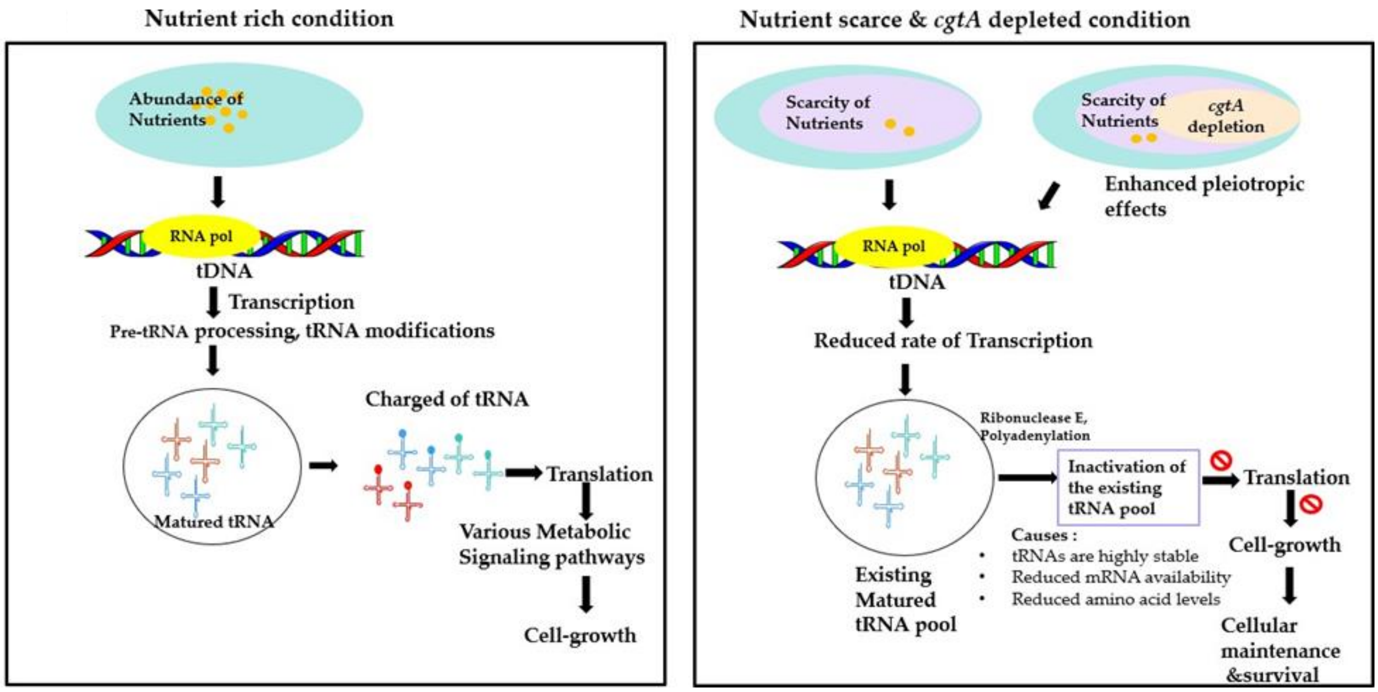
Nutrient rich condition
Abundance of Nutrients
RNA pol
tDNA
Transcription
Pre-tRNA processing, tRNA modifications
Charged of tRNA
Matured tRNA
Translation
Various Metabolic Signaling pathways
Cell-growth
Nutrient scarce & cgtA depleted condition
Scarcity of Nutrients
Scarcity of Nutrients
cgtA depletion
Enhanced pleiotropic effects
RNA pol
tDNA
Reduced rate of Transcription
Ribonuclease E, Polyadenylation
Inactivation of the existing tRNA pool
Translation
Cell-growth
Causes :
tRNAs are highly stable
Reduced mRNA availability
Reduced amino acid levels
Existing Matured tRNA pool
Cellular maintenance &survival

Fig. S6

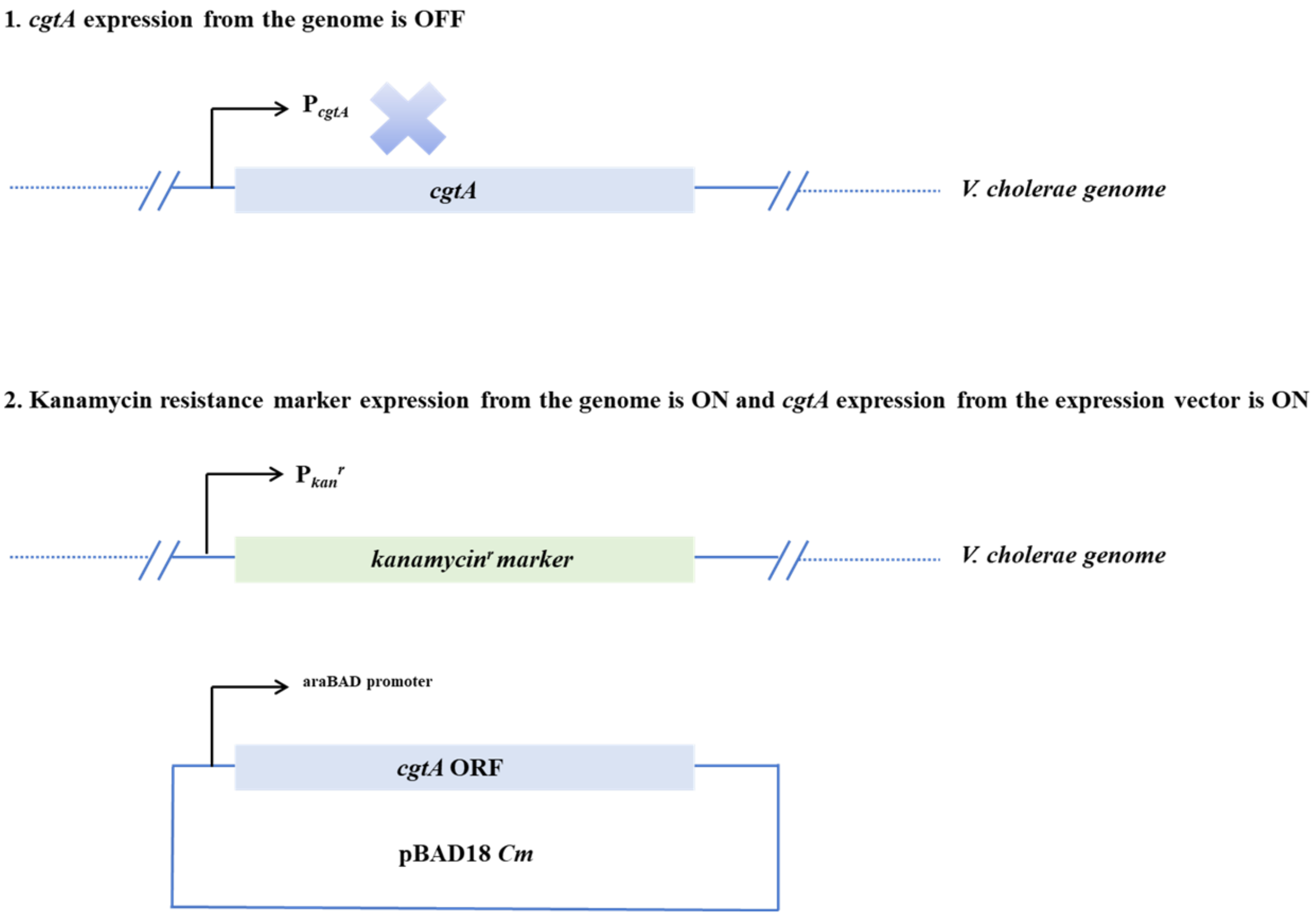

Depiction of the extra-chromosomal expression of cgtA and chromosomal expression of kanamycin marker

Fig. S7

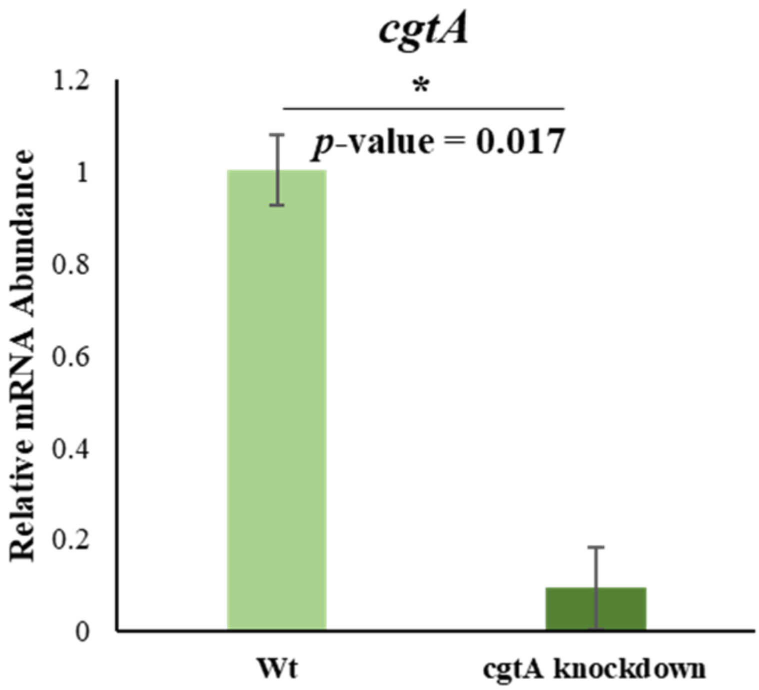

Relative *cgtA* mRNA abundance in Wt and full-length *cgtA* knockdown condition

Fig. S8

Strain name	Arabinose added	Control or Test
Wild type *V. cholerae* N16961/*cgtA*-pBAD18Cm	No	Negative control 1
Wild type *V. cholerae* N16961/ *cgtA*-pBAD18Cm	Yes	Test
Wildtype *E. coli* Top 10 / *cgtA*-pBAD18Cm	No	Negative control 2
Wildtype *E. coli* Top 10/ *cgtA*-pBAD18Cm	Yes	Positive control

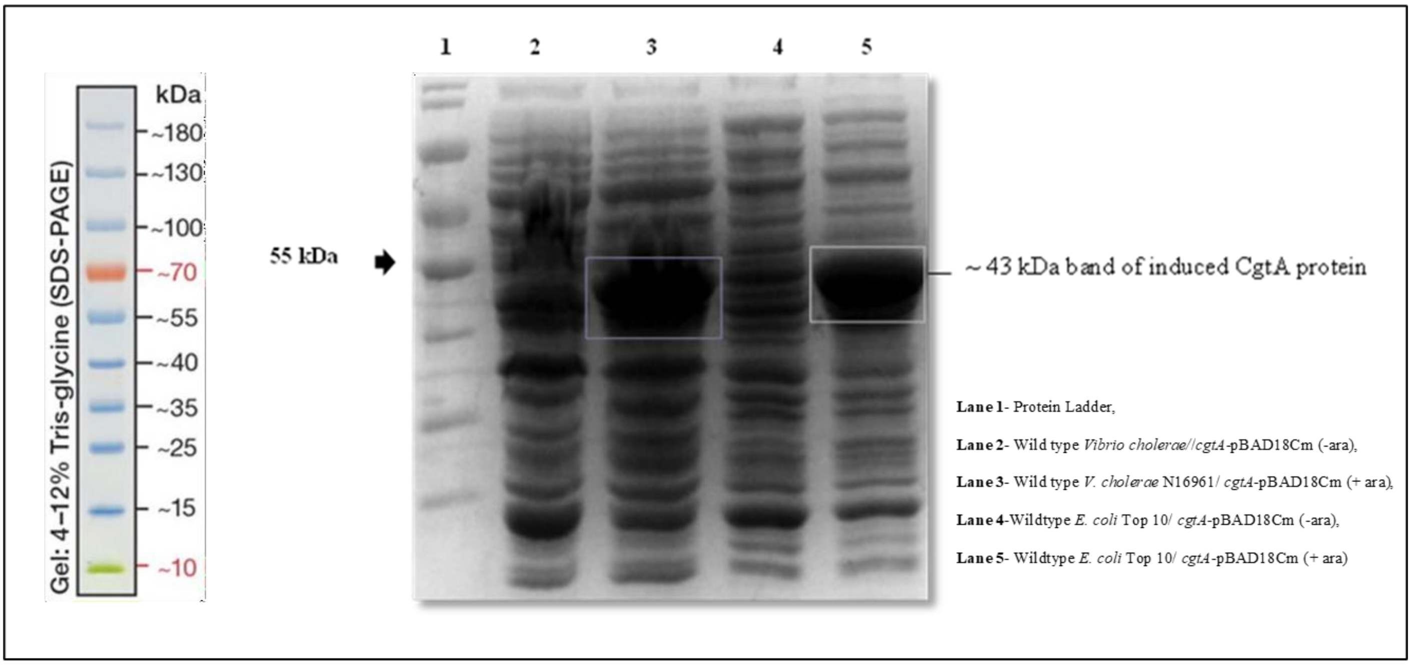

SDS PAGE analysis of the successful induction of CgtA from pBAD18Cm vector with arabinose inducer.
